# Systematic review of the use of “magnitude-based inference” in sports science and medicine

**DOI:** 10.1371/journal.pone.0235318

**Published:** 2020-06-26

**Authors:** Keith R. Lohse, Kristin L. Sainani, J. Andrew Taylor, Michael L. Butson, Emma J. Knight, Andrew J. Vickers

**Affiliations:** 1 Department of Health, Kinesiology, and Recreation, University of Utah, Salt Lake City, Utah, United States of America; 2 Department of Physical Therapy and Athletic Training, University of Utah, Salt Lake City, Utah, United States of America; 3 Department of Epidemiology and Population Health, Stanford University, Stanford, California, United States of America; 4 Physical Medicine and Rehabilitation, Harvard Medical School, Boston, Massachusetts, United States of America; 5 Swinburne University of Technology, Melbourne, Australia; 6 School of Public Health, University of Adelaide, Adelaide, Australia; 7 Department of Epidemiology and Biostatistics, Memorial Sloan Kettering Cancer Center, New York, New York, United States of America; Institute for Quality and Efficienty in Health Care (IQWiG), GERMANY

## Abstract

Magnitude-based inference (MBI) is a controversial statistical method that has been used in hundreds of papers in sports science despite criticism from statisticians. To better understand how this method has been applied in practice, we systematically reviewed 232 papers that used MBI. We extracted data on study design, sample size, and choice of MBI settings and parameters. Median sample size was 10 per group (interquartile range, IQR: 8–15) for multi-group studies and 14 (IQR: 10–24) for single-group studies; few studies reported *a priori* sample size calculations (15%). Authors predominantly applied MBI’s default settings and chose “mechanistic/non-clinical” rather than “clinical” MBI even when testing clinical interventions (only 16 studies out of 232 used clinical MBI). Using these data, we can estimate the Type I error rates for the typical MBI study. Authors frequently made dichotomous claims about effects based on the MBI criterion of a “likely” effect and sometimes based on the MBI criterion of a “possible” effect. When the sample size is n = 8 to 15 per group, these inferences have Type I error rates of 12%-22% and 22%-45%, respectively. High Type I error rates were compounded by multiple testing: Authors reported results from a median of 30 tests related to outcomes; and few studies specified a primary outcome (14%). We conclude that MBI has promoted small studies, promulgated a “black box” approach to statistics, and led to numerous papers where the conclusions are not supported by the data. Amidst debates over the role of p-values and significance testing in science, MBI also provides an important natural experiment: we find no evidence that moving researchers away from p-values or null hypothesis significance testing makes them less prone to dichotomization or over-interpretation of findings.

## 1. Introduction

Magnitude-based inference (MBI) is a controversial statistical method that has been used in hundreds of papers in the sports science and medicine literature. MBI has laudable goals. The method arose out of a desire to address over-reliance on tests of statistical significance, inattention to estimation, and distortion from unpublished null results [1]. MBI was also developed specifically for use in sports science research, where it may be difficult to get large enough sample sizes to achieve traditional statistical benchmarks such as a power of 80% at an alpha of 0.05. It is indeed useful for researchers to recognize that other choices may be justifiable, and we appreciate that MBI may have helped researchers to move beyond a narrow focus on p < .05.

However, the development and dissemination of MBI have been unusual for a statistical method. MBI has never been published in a journal following peer review by statisticians, and equations with formal notation have never been provided. In fact, since its introduction into the peer-reviewed literature in 2006 [1], the method has been criticized by statisticians [2–8]. Specifically, critics have noted that MBI lacks a clear mathematical foundation, conflates frequentist and Bayesian statistical methods; provides inappropriate sample size calculators that may underestimate sample size needs; and provides inadequate control of Type I error rates [2–8].

These critiques have led some sports science journals to caution against the use of MBI or stop accepting MBI papers altogether [9,10]; and has also led to the method receiving unfavorable media attention [11–13]. Yet, MBI continues to be defended [14–17] and used (e.g., as of May 7, 2020, a Google Scholar search returned 350 hits for “magnitude based inferences” since January 1^st^, 2019), thus necessitating further scrutiny of the method. We note that in late 2019, magnitude-based inference was renamed to magnitude-based decisions (MBD) [18]. We use the method’s original name because the studies we examined were published before the name change occurred; and we find that most published studies continue to use the term MBI (e.g., as of May 7, 2020, a Google Scholar search returned 55 hits for “magnitude based inferences” and only 17 hits for “magnitude based decisions” since January 1^st^, 2020).

To date, reviews of the method have focused on theoretical issues [2–8] and have not systematically evaluated how the method is being used in practice. This is a particularly important gap as the Type I error rates associated with MBI are highly dependent on how the method is applied [5]. Evaluating the practical application of MBI is also important in addressing proponents’ claims about MBI [14–17], including that it represents an advancement over standard null hypothesis significance testing and that it has better Type I *and* Type II error rates than null hypothesis significance testing. Finally, an empirical investigation of MBI may have broader lessons for the statistical community. Many commentators have suggested that moving applied researchers away from significance testing and p-values will help them to draw more rational conclusions from their data. MBI provides a natural experiment to test this hypothesis.

To evaluate MBI empirically, we undertook a systematic review of 232 sports science and medicine studies that used MBI. We aimed to document the typical use case of MBI, including: (1) study design; (2) sample size; (3) the number of dependent variables and statistical tests related to the study’s main hypotheses; and (4) the choice of MBI-specific settings and parameters. We used these data to estimate the Type I error rates associated with the typical MBI study. We also made qualitative observations about the statistical content and conclusions of these studies.

## 2. Methods

### 2.1. Eligibility criteria

We included all Populations, Interventions, Control groups, or types of Outcomes in our search so that our sample should be broadly representative of how magnitude-based inference (MBI) is applied in practice [19].

### 2.2. Information sources and search strategy

A systematic search was undertaken on August 2nd, 2018 in the PubMed and SportDiscus (EBSCO) databases. Searches combined the term “magnitude based inference” with “sport” or “exercise” to narrow the focus to those articles most relevant to sport and exercise science. On SportDiscus, e.g., [“magnitude based inference” AND (“sport*” OR “exercise”)]. A wild-card symbol (*) was used with “sport” to capture variations on that word. Searches included August 2nd 2018 to the earliest available date.

### 2.3. Study selection

**Fig 1:**
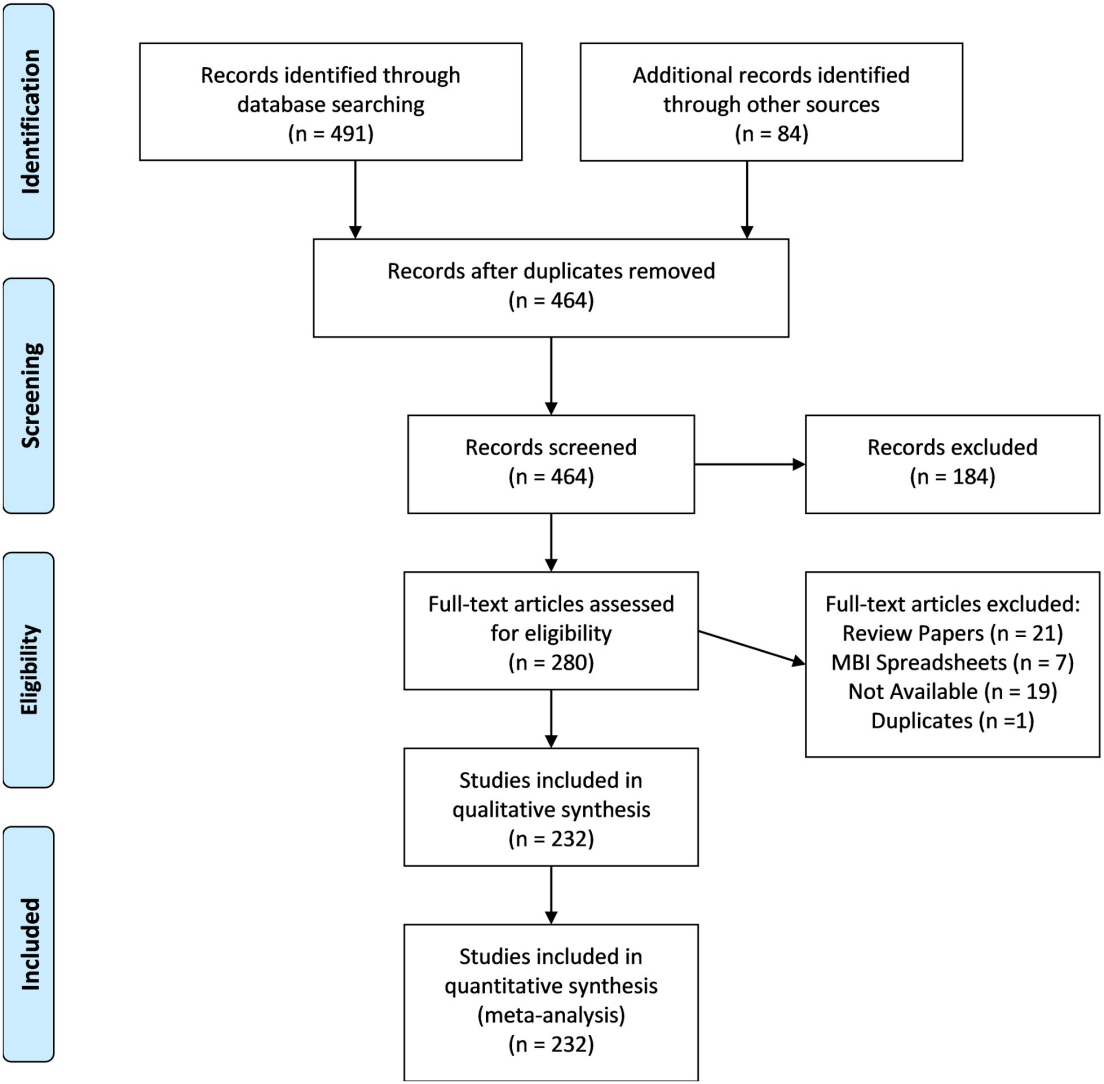
PRISMA flowchart. PRISMA flowchart showing the screening of articles through the systematic review process.

The resulting 491 results were combined with 84 results identified in a previous search [6], see the PRISMA flow diagram [20] in Fig 1. Articles were screened by title and abstract, and then full-text by KRL and a student assistant. Studies were included if they reported original empirical data (i.e., no meta-analyses, review articles) on human subjects. A total of 232 articles were included in the review (Fig 1).

### 2.4. Data collection and data items

All authors assisted in the development of a data extraction tool that went through 4 rounds of revision before being converted into a REDCap^®^ survey hosted at the University of Utah. A re-creation of the data extraction tool is shown in S1 Appendix. Variables included journal, study design, sample size, the number of dependent variables related to the main hypothesis, the number of statistical tests run pertaining to the main hypothesis, the inclusion of an *a priori* power calculation, information related to bias, and the choice of MBI settings and parameters (which are described in more detail below).

Articles were randomly assigned to random pairs of five of the authors (KRL, EJK, MLB, JAT, KLS) and each author entered the relevant data into REDCap. When data extraction was complete, the full dataset was downloaded and agreement between authors was calculated. Percentage agreement was moderate to high across items, median = 77%, IQR = [65%, 88%]. A third reviewer (KRL) then conducted consensus coding for all entries on all items in which there was disagreement. Remaining points of disagreement or areas of ambiguity where discussed with a fourth reviewer (KLS). Both the third and fourth reviewer were blind to the identities of the initial raters. See S1 Appendix for more details.

### 2.5. Risk of bias in individual studies

Selection, performance, and detection bias were assessed for individual studies. Details of this assessment are presented in S1 Appendix.

### 2.6. MBI description, settings, and parameters

MBI is implemented in downloadable Excel spreadsheets available at the website sportsci.org [18]. Though a detailed mathematical description has never been provided by the method’s developers, the statisticians Alan Welsh and Emma Knight published a statistical critique of MBI in 2015 in which they reverse engineered the formulas from the spreadsheets for the problem of comparing two means [3]. MBI involves several settings and parameters (defined below). Defaults for the parameters are provided in the spreadsheets.

MBI practitioners define a trivial range of effects that they would consider clinically irrelevant. The thresholds for harm and benefit—which we will denote −*δ*_*h*_ and *δ*_*b*_, respectively—define this trivial range. The default setting for the trivial range is −*δ*_*h*_ = −0.20 to *δ*_*b*_ = +0.20 standard deviations [18].

MBI returns probabilities that an effect is negative (or harmful), trivial, or positive (or beneficial). These probabilities are based on interpreting p-values from two one-sided hypothesis tests as if they were Bayesian posterior probabilities [3,8]. For example, if p = .25 for the null hypothesis that the intervention is not beneficial (H_0_: true effect ≤ *δ*_*b*_), MBI would conclude that there is a 25% chance that the intervention is not beneficial and thus a 75% chance that the intervention is beneficial. MBI practitioners choose parameters called the maximum risk of harm and minimum chance of benefit—which we will denote *η*_1*h*_ and *η*_1*b*_. An effect is deemed “unclear” when the harm and benefit probabilities exceed *η*_1*h*_ and *η*_1*b*_, respectively. Otherwise, an effect is deemed “clear.”

When an effect is deemed clear, MBI returns a categorical inference such as “possibly positive,” “likely positive,” or “very likely positive.” These categories are determined by the MBI probabilities: ≤0.5% is most unlikely; 0.5–5% is very unlikely; 5–25% is unlikely; 25–75% is possible; 75–95% is likely; 95–99.5% is very likely; ≥99.5% is most likely or almost certainly [1,18,21]. For example, a beneficial/positive probability of 75% to 95% corresponds to “likely positive.” This is equivalent to rejecting the null hypothesis that the effect is not beneficial/positive (H_0_: true effect ≤ *δ*_*b*_) at a significance level of .25 but not .05. A “possibly positive” result is equivalent to rejecting H_0_: true effect ≤ *δ*_*b*_ at a significance level of .75 but not .25.

MBI is divided into two types of inferences—“clinical” and “non-clinical/mechanistic”; correspondingly, the MBI spreadsheets return two sets of results, one for clinical MBI and one for non-clinical MBI. There are two main differences: (1) clinical MBI allows different values for *η*_1*b*_ and *η*_1*h*_, whereas non-clinical MBI treats both directions equivalently, such that *η*_1*b*_ = *η*_1*h*_, and (2) clinical and non-clinical MBI have different default values—0.5% and 5%, respectively—for *η*_1*h*_ (the “maximum risk of harm”). The choice of when to use clinical MBI versus non-clinical MBI is not clearly defined and a source of confusion in MBI [3]. As we will show later, the vast majority of MBI practitioners report the non-clinical/mechanistic MBI results even when reporting about interventions that affect human health or performance. We note that the MBI spreadsheets also return a third set of results called the “odds ratio interpretation” but this was only reported in nine papers that we reviewed, and the authors indicated that this option gave similar results to standard clinical MBI.

**Fig 2:**
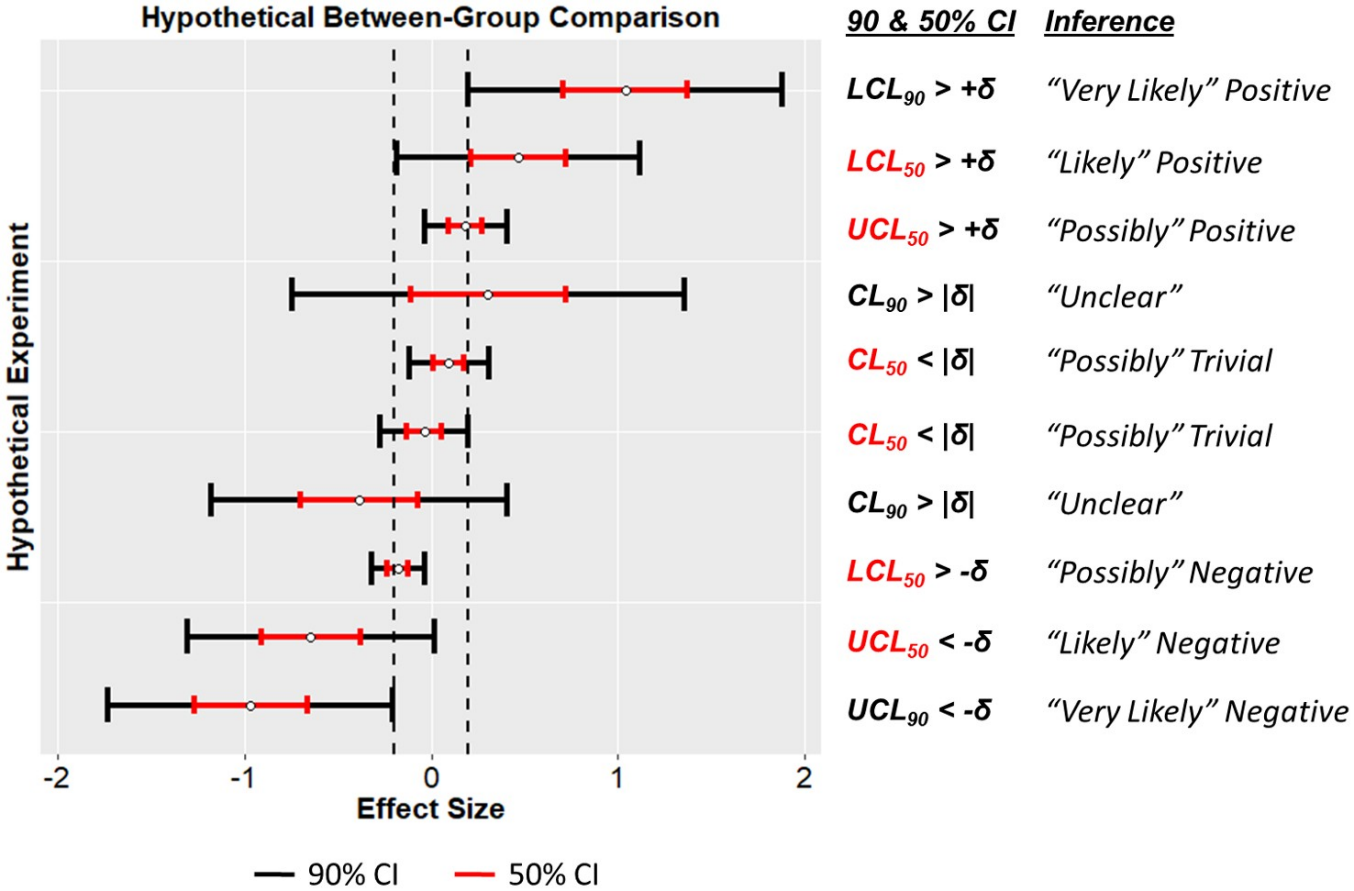
Example MBI inferences. Ten hypothetical results and corresponding MBI inferences, assuming: a trivial range of -0.2 to 0.2 standard deviations, maximum risk of harm of 5%, and equivalent treatment of positive and negative directions (non-clinical MBI). MBI inferences correspond to the locations of the 50% and 90% confidence intervals relative to the negative (or harmful), positive (or beneficial), and trivial ranges. The result is deemed “unclear” if the 90% confidence interval spans the trivial range. Of note, minimal effect testing with α = 0.05, two-sided, would not arrive at conclusions of negative or positive for any of the examples shown. Equivalence testing with α = 0.05 would also fail to conclude equivalent (i.e., trivial difference) for any of the examples shown.

Fig 2 gives several examples of how inferences are called in non-clinical MBI. In non-clinical MBI, using the default values of *η*_1*h*_ = *η*_1*b*_ = 5%, an effect is deemed “unclear” if the harm and benefit probabilities both exceed 5%; this is equivalent to the 90% confidence interval for an effect overlapping both the positive (or beneficial) and negative (or harmful) ranges (Fig 2). The default *η*_1*h*_ for clinical MBI (0.5%) is much more stringent than for non-clinical MBI (5%), which means that it is harder to find “clear” positive effects with clinical MBI than with non-clinical MBI (at default values). When an observed effect is in the positive direction, it will be declared “unclear” in clinical MBI but “clear” in non-clinical MBI if the 99% confidence interval overlaps the negative range but the 90% confidence interval does not. We speculate that this is why most users report the non-clinical MBI results.

### 2.7. Calculation of type I error rates

MBI inferences are based on one-sided hypothesis tests with significance levels of .005, .05, .25, and .75. For example, a “very likely positive” result is achieved when one rejects the null hypothesis of not beneficial/positive (H_0_: true effect ≤ *δ*_*b*_) at a significance level of .05; a “likely positive” result is achieved when one rejects the null hypothesis of not positive at a significance level of .25 (but not .05); and a “possibly positive” result is achieved when one rejects the null hypothesis of not positive at a significance level of .75 (but not .25). Thus, one might expect that the Type I error rates for these inferences would equal these significance levels. However, the calculation of Type I errors is complicated because otherwise beneficial/positive results may sometimes be deemed “unclear”, which can lower the Type I error rate. Whether results are clear or unclear is affected by the standard error (which depends on the statistical comparison of interest, outcome variance, and sample size) and the choice of MBI parameters. Therefore, unlike standard hypothesis testing, the Type I error rate for MBI depends on the sample size, study design, outcome variance, and the choice of MBI parameters. Additionally, the Type I error rates depend on the size of the true effect (which may be anywhere in the trivial range), and must account for errors in both directions (for non-clinical MBI).

We simulated the Type I error rates for MBI for both a between-group comparison and a within-group comparison assuming a range of sample sizes. For the between-group comparison, we generated 200,000 simulated trials with *n* per group from two normally distributed populations with the same variance and zero or a trivial difference between the groups. For the within-group comparison, we generated 200,000 simulated trials with a sample size of n from a normally distributed population with a true effect size of 0 or a trivial effect size. Type I error rates were then calculated as the percentage of studies in which MBI returned a positive or negative inference that met a given minimum evidence threshold (e.g., “likely”) or the percentage of studies where p < .05 (for standard hypothesis testing). Simulations were conducted in SAS 9.4. See S2 Appendix for the simulation code, which is given in both SAS and R.

We also calculated the Type I error rates mathematically, using previously derived mathematical equations for the Type I error rate [5]. These equations were written for clinical MBI; we adapted these to encompass non-clinical MBI and also provided an exact rather than approximate solution. See S1 Appendix for more details. See S2 Appendix for SAS and R code that implements the math equations. Simulated and math-predicted results matched exactly, as shown in S3 Fig of S1 Appendix.

We calculated Type I error rates for a range of scenarios. All reported values were confirmed by both simulation and math. For the base-case, we assumed *η*_1*h*_ = 5%; thresholds of harm/benefit of 0.2 standard deviations; and a true effect size of 0, to facilitate comparison with standard hypothesis testing. We assumed variances of either 1.0 (as in a cross-sectional study) or 0.364 (as might arise in a pre-post parallel trial design in which the within-person variance is lower than the between-person variance). We then varied these parameters.

## 3. Results

Our systematic review identified 232 studies that used MBI. The topics covered in these articles spanned the range of sport and exercise science and were published in highly cited journals in these fields, see Table 1.

**Table 1:** The top five most frequent venues for MBI publications identified in our review.

Journal Title	Journal Impact Factor	Number of MBI Publications
The Journal of Strength and Conditioning Research	2.325	35
The International Journal of Sports Physiology and Performance	3.384	24
Journal of Sports Sciences	2.733	17
PLoS One	2.766	11
Frontiers in Physiology	3.394	10

Journal impact factors were extracted from the Journal Citation Reports database on 2019-3-20.

The studies covered a range of experimental designs, including randomized controlled trials, crossover trials, and observational studies, see Table 2. Sample sizes were extremely small: The median sample size was 10 per group (interquartile range: 8 to 15 per group) for multi-group studies and 14 (interquartile range: 10 to 24) for single-group studies. Only 15% of studies reported any *a priori* power analysis. In cases where authors reported sample size calculations based on the MBI calculators, they often reported very low sample size requirements, such as 5, 6, or 7 total participants [22–24]. We also note that many authors seemed to erroneously believe that use of MBI circumvents the need for an adequate sample size [25–29]. For example, Stanley et al. [25] wrote of MBI: “With this statistical approach, our sample size is not limiting.”

**Table 2:** Descriptive statistics of the 232 articles identified in the systematic review, median [IQR] or N (%). MBI settings were not discernible from all studies, as indicated.

Measure	Median [IQR] or N(%)
N per group for studies with >1 group (n = 111)^a^	10 [8, 15]
Total N for single group studies (n = 121)	14 [10, 24]
Number of dependent variables	7 [5, 12]
Number of Statistical Tests pertaining to the main hypotheses	30 [15, 56]
MBI Parameters^b^	
Harm/negative Threshold = -0.2	182 (79%)
Benefit/positive Threshold = +0.2	181 (78%)
maximum risk of harm (*η*_1*h*_) = 5%	183 (79%)
Statement of *a Priori* Power Calculation	34 (15%)
‘Primary’ Variable Explicitly Defined	33 (14%)
Attrition or Exclusions Stated	55 (24%)
Described as Bayesian	0 (0%)
NHST also Performed	108 (47%)
Minimum MBI evidence threshold applied^c^	
“Possible” (≥25%)	88 (38%)
≥50%	19 (8%)
“Likely” (≥75%)	100 (43%)
“Very likely” (≥95%)	0 (0%)
Not able to be determined	25 (11%)
Study Design	
RCT	53 (23%)
Cross-Over	58 (25%)
Observational	95 (41%)
Other	26 (11%)
Clinical or Non-Clinical MBI	
Clinical, explicitly stated	8 (3.4%)
Non-clinical, explicitly stated	37 (16%)
Both, explicitly stated	3 (1.3%)
Determined to be clinical though not explicitly stated^d^	5 (2.2%)
Determined to be non-clinical though not explicitly stated^e^	164 (71%)
Not able to be determined	15 (6.1%)

^a^Of these, 72 were two-group studies. The median [IQR] of sample size for two-group studies was: 10 [8,14].

^b^Our counts may represent an underestimate of the number of times the default MBI parameters were used, as some papers provided insufficient information to determine these values. We were unable to discern a value for the harm/negative threshold in 35 papers, the benefit/positive threshold in 32 papers, and the maximum risk of harm in 20 papers.

^c^Some authors explicitly set a minimum evidence threshold above which effects were declared “implementable”, “substantial”, or “practically meaningful.” Others implicitly set this threshold by only choosing to highlight and draw conclusions based on effects that met a given evidentiary threshold, such as “likely” or “possible.”

^d^Clinical MBI was inferred from statements such as: “a clinically clear beneficial effect was at least possibly beneficial (>25% chance) and almost certainly not harmful (<0.5% risk).” Our count includes one paper that was explicitly labeled as non-clinical MBI but we believe to have run clinical MBI.

^e^Non-clinical MBI was inferred from the statement: “When the positive and negative values were both >5%, the inference was classified as unclear” or, equivalently, “If the 90% confidence interval overlapped the thresholds for the smallest worthwhile positive and negative effects, effects were classified as unclear.” In a few other cases, non-clinical MBI was determined mathematically based on how “unclear” results were called. Our count includes two papers that were explicitly labeled as clinical MBI but we believe to have run non-clinical MBI.

Focusing solely on analyses related to the main hypotheses (excluding baseline and other ancillary comparisons), the median number of dependent variables was 7 and the median number of statistical tests run was 30. Despite this multiplicity of outcomes, few studies (14%) defined one or more variables as “primary.”

The vast majority of studies used non-clinical MBI with the same values: trivial thresholds between -0.20 and +0.20 SD with *η*_1*h*_ = 5%. Authors explicitly noted the use of clinical MBI (3.4%), non-clinical MBI (16%) or both (1.3%) in a minority of papers. Given descriptions in the texts, we identified five additional papers where we believe clinical MBI was used, for a total of 16 papers (7%) that used clinical MBI. We believe that non-clinical MBI was used in almost all other cases (164 additional papers). In these cases, authors wrote a version of the following statements: “When the positive and negative values were both >5%, the inference was classified as unclear” or, equivalently, “If the 90% confidence interval overlapped the thresholds for the smallest worthwhile positive and negative effects, effects were classified as unclear”; this means they used *η*_1*h*_ = 5% and treated both directions equivalently, consistent with non-clinical MBI. We cannot rule out that authors used clinical MBI with both *η*_1*h*_ = 5% and *η*_1*b*_ = 5%, but this is less likely given that these are not the usual default choices for clinical MBI.

MBI provides categories of evidence, such as “possible”, “likely”, and “very likely” beneficial. We found that researchers employed MBI’s evidence thresholds much like significance thresholds: They highlighted results that met the bars of “possible”, “likely”, or higher in tables and figures with symbols to distinguish the achieved thresholds much like the use of symbols for p<0.05 or p<0.01; for examples, see: [30–32]. They then made claims about effects based on meeting a minimum MBI evidence threshold, usually either “possible” or “likely.” For example, Cruz et al. [33] explicitly define this bar, declaring: “A likely difference (>75%) was considered as the minimum threshold to detect meaningful differences,” as do Weaving et al. [34]: “The magnitude of difference was considered practically meaningful when the likelihood was ≥ 75%.” Others implicitly set a minimum threshold by only highlighting and drawing conclusions based on effects above a certain bar. For example, Lahart et al. [24] write in their abstract: “Magnitude-based inference analyses revealed **likely** at least small beneficial effects (effect sizes≥.20) on absolute and relative VO_2_max (d = .44 and .40, respectively), and total and moderate PA (d = .73 and .59, respectively) in the intervention compared to the usual care group. We found no **likely** beneficial improvements in any other outcome” (emphasis added). One can infer from their description and ensuing positive recommendation (“This intervention has the potential for widespread implementation and adoption, which could considerably impact on post-treatment recovery in this population.”) that they used “likely” as their minimum evidence threshold, though this was never explicitly stated.

We found that most authors used a minimum evidence threshold of “possible” (38%) or “likely” (43%) (Table 2). We found no cases where authors either explicitly or implicitly set this bar higher than “likely” (Table 2). In fact, authors appeared to view the “likely” threshold as a high level of evidence. For example, Barrett et al. [35] state: “We adopted a **conservative approach to inference** whereby substantial effects were only declared clear when the probability likelihood for the effect was ≥75% (i.e., likely)” (emphasis added).

Almost half (47%) of studies performed null hypothesis testing alongside MBI. Most authors reported using the Excel spreadsheets to run their MBI analyses, but a few authors reported running frequentist statistical models in other programs, such as SAS, but then interpreted the resulting frequentist confidence intervals in MBI terms. We found no studies out of 232 that described their statistical approach as Bayesian.

S1 Appendix documents additional concerns about bias in the design and reporting of these studies. However, we note that these concerns are common in sports science studies and are not unique to studies using MBI.

In our review, we found that most MBI studies employed a narrow range of sample sizes, used non-clinical MBI, and used the same default MBI settings: thresholds for harm/benefit of 0.2 and maximum risk of harm of 0.05. Thus, we were able to estimate the Type I error rates that apply to the vast majority of the MBI literature. We estimated the Type I error rates associated with the “possible” and “likely” evidence thresholds since, in the studies we reviewed, authors consistently highlighted and made claims based on “possible” or “likely” positive/beneficial or negative/harmful inferences. Note that our estimates of Type I error would also apply to clinical MBI with *η*_1*b*_ = *η*_1*h*_ = 5%. In fact, clinical MBI with *η*_1*b*_ = *η*_1*h*_ = 5% (rather than *η*_1*b*_ = 25% and *η*_1*h*_ = 0.5%) was given as the default choice in at least one version of the MBI spreadsheets that we found online in August of 2019 (see S3 Appendix; [18]).

**Fig 3:**
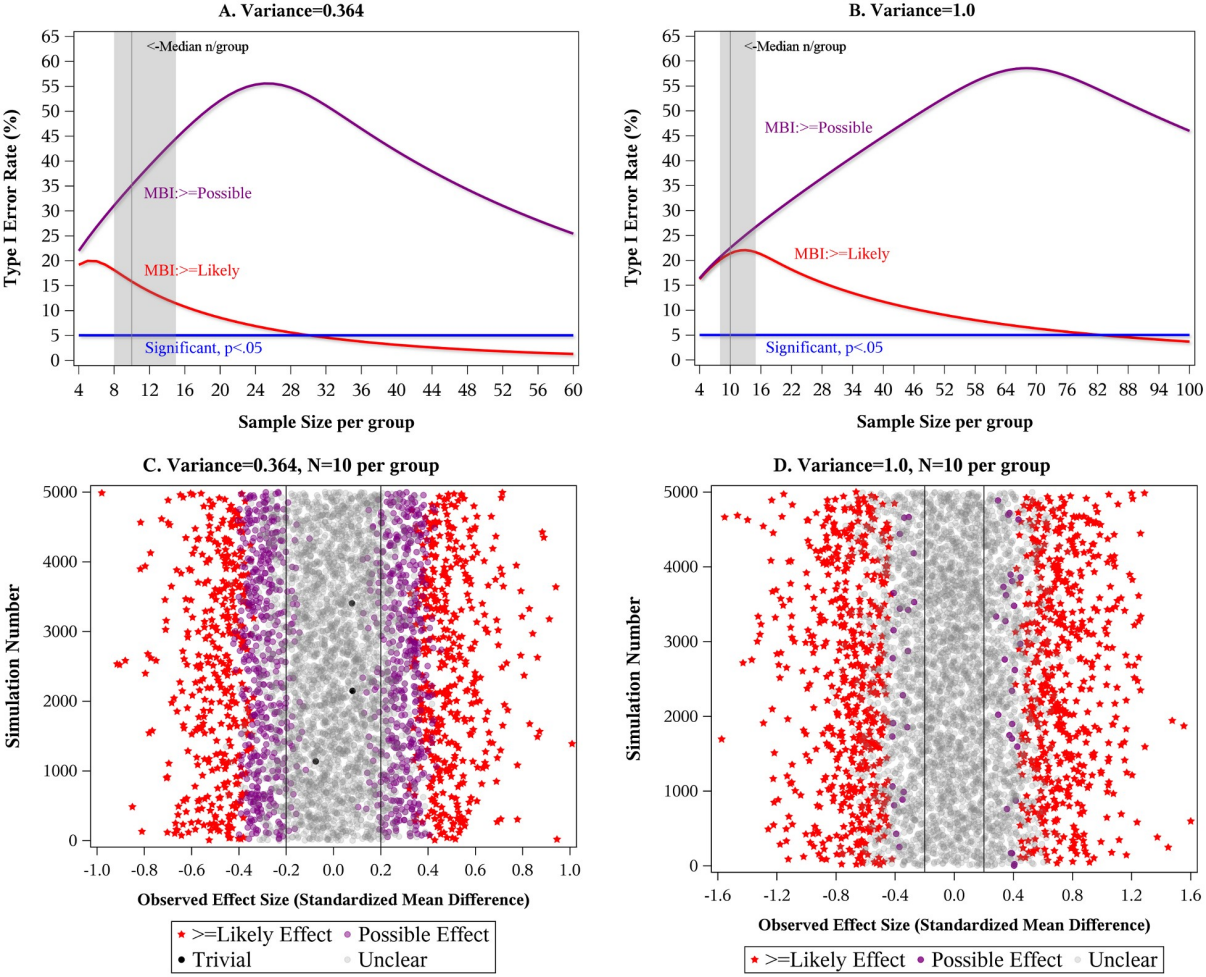
MBI’s Type I error rates. A and B: Type I error rates for MBI’s “possible” (purple) and “likely” (red) thresholds, as well as standard hypothesis testing at α = 0.05 (blue) as a function of sample size. The statistical comparison is a two-group comparison of means. True effect size = 0, meaning there is no difference between the groups. (A) assumes variance of 0.364, as might arise in a pre-post study, whereas (B) assumes a variance of 1.0, as in a cross-sectional study. Shaded area shows the interquartile range of sample sizes of the reviewed studies; vertical reference line is the median sample size. Type I error rates were identical whether calculated mathematically or by simulation with 200,000 repeats (see S1 Appendix). C: MBI results from 5000 simulated trials where variance = 0.364 and n = 10 per group. D: MBI results from 5000 simulated trials where variance = 1.0 and n = 10 per group. Simulations and calculations use the MBI settings that predominate in the literature: trivial range of -0.2 to 0.2; maximum risk of harm of 5%; and equivalent treatment of positive and negative directions.

Fig 3A and 3B show the Type I error rates as a function of sample size for a two-group comparison of means when the outcome variance is smaller (3A) or larger (3B); patterns are similar for other statistical comparisons (see S1 Appendix). MBI creates pockets of high Type I error that—particularly for the “likely” threshold—coincide with the sample sizes that predominate in the MBI literature. Fig 3C and 3D demonstrate how easy it is for spurious “likely” effects to arise in small samples due to high chance fluctuation.

**Fig 4:**
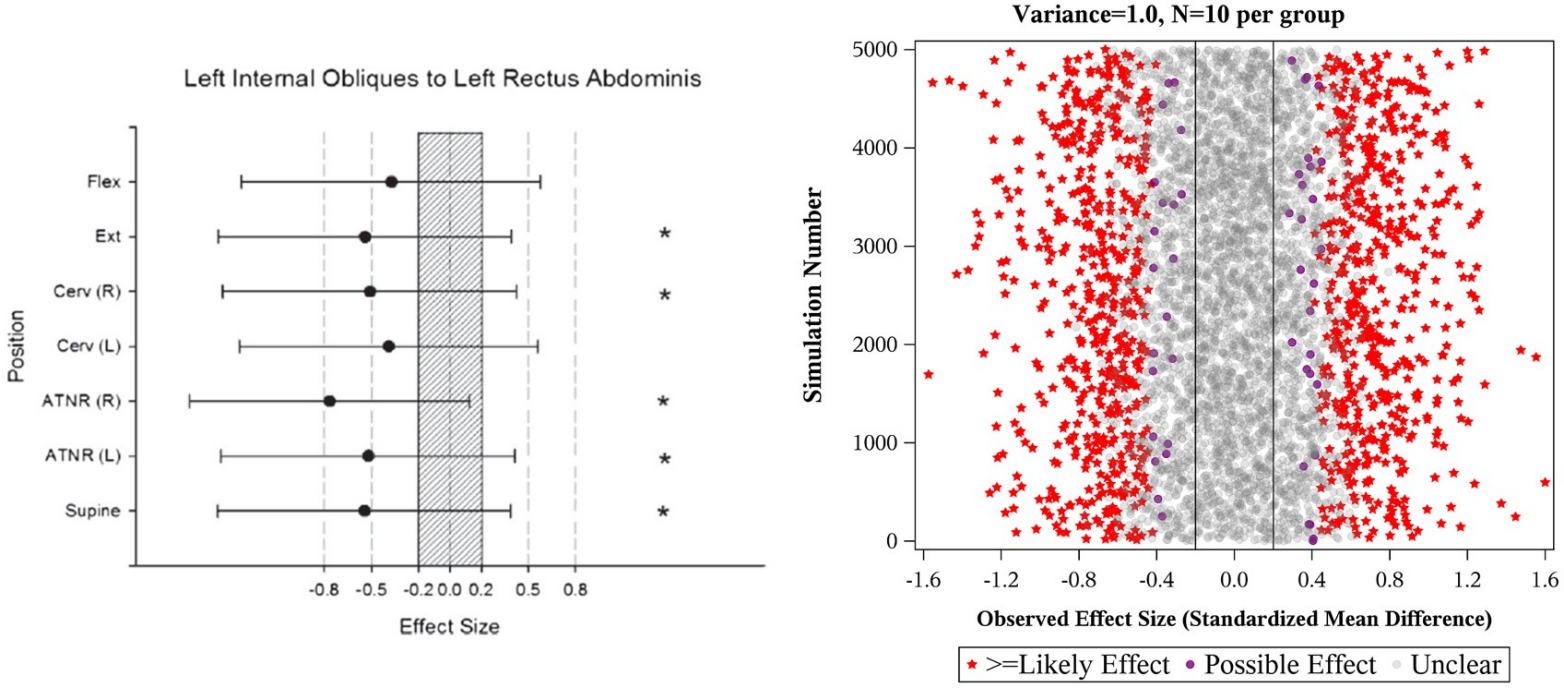
An example of MBI inferences in practice. Left Panel (Reproduced from Parfey et al. [50], Fig 2C): Literature example where effects deemed “likely” by MBI are associated with large p-values. Confidence intervals are 95% CIs. Starred values are effects meeting MBI’s “likely” threshold. These results were interpreted as evidence of a difference between groups; the authors concluded: “Individuals with CLBP and PR manifested altered activation patterns during the hollowing maneuver compared to healthy controls.” Right panel: Simulation that shows the MBI inferences that are expected for a study of this type (n = 10 per group, cross-sectional) when the true effect is 0. Note that in both the real example and the simulation, most observed effects larger than 0.5 are deemed “likely”.

For multi-group studies, the interquartile range of sample size was 8 to 15 per group. At these sample sizes, the Type I error rate for the “possible” threshold is 22% to 45% and for the “likely” threshold is 12% to 22% (Fig 3A and 3B). Consistent with these calculations, we noticed numerous studies in which effects associated with large p-values (for H_o_ = 0)—often in the 0.2 to 0.3 range—were declared “likely” or higher by MBI [36–52]. See Fig 4 for an example from the literature.

In some examples, high p-values were reported alongside the MBI results. When p-values were reported alongside MBI results, we found that MBI descriptors were frequently prioritized above p-values in the interpretation of results. Indeed, the results were often used to justify the superiority of MBI over conventional approaches. Examples include:

“Although parametric analysis was unable to demonstrate significant differences, magnitude-based inferences indicated that the change in 1-RM squat showed a likely benefit from PA on increasing lower body strength and a very likely benefit for increasing lean body mass (LBM).”

(Hoffman et al. [43])

“Because a repeated measures ANOVA could not detect any significant difference in the variables between the fastest and slowest trials (P = .136 –>.999), magnitude-based inference approach was adopted to analyse the differences at each step.”

(Nagahara et al. [44])

“There were no statistically significant differences between the non-runners and runners groups in the CRP values for any of the coupling pairs analysed, however, the magnitude-based inferences enabled more precise tracking of differences between groups and some of the differences were substantially clear.”

(Lockie et al. [45])

“There were no statistical differences in NB between groups (P = 0.23); however, magnitude-based inferential statistics revealed likely small (mean effect±90% CI; 0.59±0.87) and moderate (0.80 ± 0.91) increases in NB for PULSE and INT compared to BOLUS and possible small increase (0.42 ± 1.00) for INT vs. PULSE. Conclusion: We conclude that the pattern of ingested protein, and not only the total daily amount, can impact whole-body protein metabolism.”

(Moore et al. [46])

In these instances, and others, the authors emphasized the more favorable MBI results in their conclusions, ignoring that the observed effects could easily have arisen due to sampling variability.

In many other examples, authors did not report p-values, but we back-calculated high p-values from confidence intervals [52]. For example, one study reported an improvement of 0.46 standard deviations in countermovement jumps with a 90% confidence interval of -0.10 to 0.91 standard deviations [52]; we calculated the two-sided p-value associated with H_o_ = 0 as: P(T_21_>(.46/(.5/1.72)))+P(T_21_<-(.46/(.5/1.72))) = .13. Notably, this paper reported that the intervention “almost certainly” improves countermovement jumps with a 100% probability of benefit [52].

Until now, our discussion has focused on a single test with default MBI parameters. Table 3 shows how changing the study design, variance, and MBI parameters affects the Type I error rates. We found that across most realistic scenarios, researchers have between a 20% to 52% chance of getting a “possible” or higher positive or negative inference when the true effect is zero or trivial. Increasing sample size or decreasing variance (relative to the base case) results in lower Type I error rates for the “likely” threshold, but higher rates for the “possible” threshold. Changing *η*_1*h*_ from 5.0% to 0.5% lowers the Type I error rates for these small sample sizes, but as previously noted, researchers rarely chose this value (n = 19, 8% of studies).

**Table 3:** Type I error rates for MBI vary as a function of sample size, statistical comparison, variance, maximum risk of harm, and thresholds for harm/benefit. Row 1 represents the typical MBI study; subsequent rows demonstrate how changing specific parameters alters the rates; parameters that remain unchanged from the base case are grayed out whereas altered parameters are bolded. Rates were calculated mathematically and also confirmed by simulation with 200,000 repeats (See S1 Appendix for description and S2 Appendix for code).

Statistical comparison	Parameters						Type I error rates			
	Sample size per group	Variance^a^	Maximum risk of harm	Threshold for harm/benefit	True trivial effect size^b^	Number of statistical tests run^c^	MBI “possible” threshold	MBI “likely” threshold	p < .05	p < .01
Two-group pre-post	10	0.36	0.05	0.2	0	1	35%	16%	5%	1%
Two-group pre-post	**20**	0.36	0.05	0.2	0	1	52%	9%	5%	1%
**Two-group cross-sectional**	10	**1.0**	0.05	0.2	0	1	22%	21%	5%	1%
**Within-group**	**14**	**0.36**	0.05	0.2	0	1	48%	6%	5%	1%
**Within-group**	**14**	**0.36**	0.05	**0.1**^d^	0	1	29%	19%	5%	1%
**Within-group**	**14**	**0.80**	0.05	0.2	0	1	39%	13%	5%	1%
Two-group pre-post	10	0.36	**0.005**	0.2	0	1	6%	6%	5%	1%
Two-group pre-post	10	0.36	0.05	**0.1**	0	1	20%	20%	5%	1%
Two-group pre-post	10	0.36	0.05	0.2	**+0.1 or -0.1**	1	38%	19%	6%	1.5%
Two-group pre-post	10	0.36	0.05	0.2	0	**10**	99%	82%	40%	10%

^a^Calculations use standardized effect sizes, so variance = 1. But for statistical comparisons that involve change scores, the within-person variance may be lower than 1.

^b^Type I error rates can also be calculated for non-zero, trivial effects.

^c^When number of statistical tests>1, the Type I error rates represent the chance of at least one false positive, calculated assuming independent tests.

^d^Some within-person studies use a threshold for harm/benefit of 0.3 of the within-subject coefficient of variation; this typically translates to a smaller trivial range than 0.2 baseline standard deviations.

MBI’s high Type I error rates are further compounded by multiple testing (Table 3). Though multiple testing is also an issue for standard hypothesis testing, the higher per-test Type I error rates of MBI serve to exacerbate the issue. The studies we reviewed reported a median of 7 dependent variables and 30 statistical tests related to the main hypothesis. With so many tests, users are virtually guaranteed to find some “possible” or higher effects by random chance when the intervention is ineffective (Table 3). MBI provides few tools for addressing multiple testing; in fact, the proponents of MBI have advised against controlling for multiple tests [21]. MBI also provides no means for handling repeated-measures data; thus, in papers with more than two time points, authors invariably presented MBI analyses for each time point separately, creating an obvious multiple testing issue. For example, in a parallel-group randomized trial, Townsend and colleagues [53] measured muscle biomarkers in an exercise and control group at 3 follow-up time points: 1 hour, 5 hours, and 48 hours. Using repeated-measures ANOVA, they found no significant time-by-group interactions for any outcome at the 0.05 level; however, the MBI analysis found two positive effects out of 15 tested (5 outcomes by 3 timepoints): a “likely” increase for exercise versus control in IkBα phosphorylation at 5 hours and a “very likely” increase for exercise versus control in total c-Myc content at 5 hours. They then concluded: “The main findings of this investigation indicated that phosphorylation of IkBα and total c-Myc content was increased in skeletal muscle following resistance exercise in novice lifters.”

## 4. Discussion

Through our systematic review, we have documented that studies using MBI are typically small in size and often make strong claims based on weak evidence. MBI authors routinely made claims based on achieving MBI inferences of “likely” and sometimes “possible” effects. These inferences carry high Type I error rates—12%-22% and 22%-45%, respectively, for the way that MBI is typically applied in the literature. MBI users predominantly applied MBI’s default settings, used a maximum risk of harm of 5%, and chose “non-clinical” rather than “clinical” MBI even when testing interventions in humans. This is likely related to “non-clinical” MBI providing stronger inferences for a given set of data as well as to a lack of precise guidelines about when to use non-clinical versus clinical MBI. Few studies reported *a priori* power calculations (15%) or specified a primary outcome (14%). Multiple testing was common with a median of 7 dependent variables and 30 tests related to the main hypotheses of interest; this exacerbates the Type I error issue.

The median sample size was 10 per group for multi-group studies and 14 total for single-group studies. As a benchmark, it is worth noting that a sample size of 10 per group has a mere 40% power to detect an effect size of 0.8, which is commonly described as a large effect (given α = 5%). Inadequate sample sizes and poor sample size planning are certainly not unique to MBI. However, we believe that the way that MBI has been advertised to the sports science community has encouraged the use of small studies. A central justification for MBI has been its ability to generate more “publication-worthy” results from under-powered studies. For example, proponents of the method state that MBI, “provides an avenue for publishing previously unpublishable effects from small samples” [14] and that, “With small studies, you get nonsignificant effects a lot, but these nonsignificant effects are often clear so the researcher can often make useful publishable conclusions for such a thing with MBI. You can get your stuff into press” [54]. Thus, it’s not surprising that MBI users predominantly employed small samples; rarely performed *a priori* sample size calculations, and even made statements suggesting that MBI circumvents the need for an adequate sample size [25–29].

Overinterpretation of statistical results is also not unique to MBI, but features of the method and its portrayal may exacerbate the problem. MBI has been described as a Bayesian procedure [14] and “definitely not” a form of hypothesis testing [15]. MBI’s developers state: “…we wish to avoid hypothesis tests of any kind, involving either the nil (zero) hypotheses or non-zero hypotheses, as in equivalence testing” [55]. Thus, it’s not surprising that users appear unaware that, underneath the hood, MBI is in fact running non-zero hypothesis tests (with liberal significance levels). MBI also misrepresents p-values as Bayesian posterior probabilities and affixes overly optimistic descriptors such as “likely” or “almost certainly” beneficial. Correspondingly, users routinely attribute higher levels of evidence to MBI inferences than the data warrant.

MBI’s proponents have repeatedly denied that MBI has elevated Type I error rates [14–17]. We have shown this to be false. MBI does not *necessarily* carry a high Type I error rate, because MBI’s error rates depend on the choice of MBI parameters and settings as well as study design and sample size. However, we found that MBI is predominantly applied in a way that results in high Type I error. For example, use of a maximum risk of harm of 0.5% keeps Type I error rates low for samples of 8 to 15 per groups, but only 8% of users chose this value. Despite conducting clinical research in human populations, authors primarily used non-clinical MBI rather than clinical MBI, likely because it has a default maximum risk of harm of 5% and thus makes it easier to find “clear” beneficial/positive effects.

There has been disagreement about what constitutes a Type I error in MBI. The developers of MBI sometimes refer to “clinical Type I error,” which they define as claiming benefit when the true effect is harmful; however, this ignores the larger source of Type I error, which is claiming benefit when the true effect is trivial [5]. The developers of MBI have also claimed that clinical MBI incurs a Type I error for a “possibly” or “likely” inference but that non-clinical MBI does not [17]. This would only be true if non-clinical MBI required a minimum actionable evidence threshold of “very likely.” It does not. Users routinely made claims about clinical interventions based on “possibly” and “likely” inferences regardless of the choice of clinical or non-clinical MBI. In fact, only 21% of users even specified whether they were using non-clinical or clinical MBI, suggesting that this distinction holds little meaning in practice.

We acknowledge that the appropriate tradeoff between Type I and Type II error may be different in sports science than in other biomedical fields, such as cancer research. Thus, there may be cases where a Type I error rate higher than 0.05 may be justified. However, we emphasize that: (1) 0.05 is itself considered a weak level of evidence [56] (2) 0.05 has undoubtedly been given undue importance in the medical literature, but this problem is not solved by setting a more liberal default threshold; (3) the Type I error rates that we have documented for MBI would be considered unusually weak levels of evidence in biomedicine. As a benchmark, p = .25 corresponds to a Shannon-information, or S-value, of 2, meaning the data provide just 2 bits of information against the null hypothesis of no effect—no more surprising than getting two heads in two coin tosses [57]; (4) high rates of multiple testing in sports science compound the issue; and (5) if researchers are going to allow a higher Type I error rate, they must be transparent about this choice and justify it *a priori*. In the MBI literature, we found no cases where MBI practitioners acknowledged higher Type I error rates, let alone justified them.

We found that the MBI spreadsheets encourage a black box approach to statistics [8]. For example, users predominantly employed the default thresholds for harm/benefit of 0.2 standard deviations presented in the MBI spreadsheets. This suggests that this choice was not based on any biological or clinical rationale and was likely not specified *a priori*. Of course, MBI is not unique in encouraging a black box approach to statistics. Many statistical programs allow users to implement statistical methods that they do not understand. However, MBI’s lack of rigorous review and documentation may exacerbate the problem [8].

The MBI spreadsheets are also not designed to handle many common statistical situations: repeated-measures or clustered data, interactions, count data, and adjustment for more than a single covariate [8]. The inability to appropriately handle repeated-measures data is particularly problematic given that repeated-measures were common in MBI studies. For instance, in a study on foam rolling [30], group differences were assessed at three different time points for 13 outcomes measures. With a more conventional ANOVA, these post-hoc tests might only be conducted following a statistically significant Group x Time interaction, or not at all. Multiple testing is also a problem for null hypothesis significance testing, but MBI’s higher per-test Type I error rates and inability to handle repeated measures compound the problem.

We acknowledge that Type I error rates for null hypothesis significance testing can also be greatly inflated due to “p-hacking,” the practice of manipulating data and analyses to produce p-values under 0.05 [58]. Though MBI does allow other researcher degrees of freedom (e.g., choice of MBI parameters and settings), MBI may be less prone to p-hacking because it does not have the same narrow focus on p < .05. Indeed, a positive outcome of MBI is that it has helped moved sports scientists away from overreliance on p < .05.

Proponents of MBI argue that small studies are unavoidable in sports science and thus that MBI is needed to ensure a path to publication for otherwise “unpublishable” null results [14]. We disagree that small studies are inevitable, and believe that we should encourage researchers to engage in sample size planning and to design better [59] and larger studies [60,61]. We also encourage the adoption of registered reports, which prevent reliance on statistical significance as a criterion for publication [62,63]. In the absence of preregistration, when researchers do run severely under-powered studies (e.g., due to logistical constraints), we do not believe that the appropriate path to publication is choosing an inferential method that misrepresents weak evidence as strong evidence. Rather, researchers can publish the results as a pilot or feasibility study [64] or report the results descriptively, avoiding inferential language [65]. Small studies—provided that are properly reported, without overstating evidence—can be useful for the design of larger trials as well as in meta-analyses. Additionally, we believe that many sports science researchers may be interested in making decisions about individual athletes rather than making inferences to wider populations, in which case the most appropriate study design may be an N-of-1 trial [66]. If practitioners are interested in seeing if an intervention is reliably beneficial for a specific athlete, it is more meaningful and more valid to adopt an N-of-1 trial than generalize from an underpowered study using a low standard of evidence for an effect. Finally, we note that when studying elite athletes, it’s possible that a relatively small sample will give adequate power due to the low variability in the population.

Proponents of MBI have additionally argued that MBI is necessary because it provides the only alternative to null hypothesis significance testing, for example writing: “Generations of statisticians before us have also criticized the null-hypothesis test, but magnitude-based inference appears to be the first practical alternative that properly takes into account the uncertainty arising from sampling variation” [15]. This argument is false; scientists are not limited to these two approaches and MBI is certainly not the first alternative to a monolithic statistical approach. Multiple other statistically sound alternatives are available. For example, MBI uses the same underlying hypothesis tests as minimal effect testing and equivalence testing/non-inferiority testing [67]. Thus, MBI practitioners could use these procedures instead [68]. Furthermore, sports scientists should understand that—when used correctly—p-values are valid tools of inference [69]. For example, Amrhein and colleague’s [70] recent headline-making call for the abandonment of significance testing did not advocate a ban on p-values, but instead encouraged scientists to use p-values correctly—by recognizing them as a continuous (rather than dichotomous) measure and as just one piece of evidence among many. Finally, many of the stated goals of MBI—such as giving a plain language interpretation of statistics that is understandable by coaches and athletes—can be validly achieved with a Bayesian analysis [4] or by careful incorporation of p-values with data on effect sizes, as well as consideration of strengths and weaknesses of the experiment and evaluation of the current literature.

The conclusions of past studies that have used MBI should be treated with skepticism. However, these papers do still contain valid information—such as means, standard deviations, and confidence intervals—that can be used to re-evaluate the studies’ conclusions. For example, one study reporting that an intervention “almost certainly” improved countermovement jumps also reported a 90% confidence interval for this improvement of -0.10 to 0.91 standard deviations [52]. It is clear from the confidence interval that the evidence of improvement is much weaker than the study’s conclusions imply. However, the standard error is calculable allowing the data from this study to be entered into a meta-analysis.

Furthermore, some MBI papers report the exact harm and benefit probabilities given by the MBI spreadsheets, which are useful because they reveal the p-values associated with specific one-sided hypothesis tests. The harm/negative probability is the p-value associated with the null hypothesis of harm (H_o_: effect size ≤-δ_h_); and subtracting this value from 1 gives the p-value associated with the null hypothesis of no harm (H_o_: effect size ≥-δ_h_). Similarly, the benefit/positive probability is the p-value associated with the null hypothesis of benefit (H_o_: effect size ≥δ_b_); and subtracting this value from 1 gives the p-value associated with the null hypothesis of no benefit (H_o_: effect size ≤δ_b_). One may use these p-values to perform equivalence testing and/or minimal effects testing [67,68]. We give a specific example of how this would be done in Table 4 using a study on foam rolling [30]. Our re-examination of these data suggest that the study’s conclusions were overly optimistic. Notably, this paper has been cited 258 times in Google Scholar (as of 2020-01-05); the first 10 citing papers that we checked all cited MacDonald and colleagues [30] as providing evidence of the benefits of foam rolling, demonstrating how overly optimistic conclusions are easily propagated through the literature and how correcting such errors is difficult once they are published.

**Table 4:** Example of how MBI results can be re-interpreted by one-sided minimal effects testing and noninferiority testing with α = .05^a^.

Dependent variable	MBI benefit probability	95% CI for the effect	MBI interpretation	P-value for the null hypothesis of no increase (H_0_: effect ≤ δ_b_)^b^	P-value for the null hypothesis of decrease (H_0_: effect ≤ -δ_h_)^c^	Re-Interpretation
Vertical jump height (cm)						
24 hours	74%	-2.14, 6.9	Possible increase	0.26	< .05	No substantial decrease
48 hours	92%	-0.22, 5.82	Substantial increase^d^	0.08	< .05	No substantial decrease
72 hours	“Unclear”	-2.82, 2.62	Unclear	0.62^e^	0.33	Inconclusive
Quadriceps passive range of motion (degrees)						
24 hours	“Unclear”	-6.18, 8.78	Unclear	0.52	0.22	Inconclusive
48 hours	90%	-0.96, 14.16	Substantial increase	0.10	< .05	No substantial decrease
72 hours	79%	-3.24, 13.14	Substantial increase	0.21	< .05	No substantial decrease

^a^This example study (MacDonald et al. [30]) was a randomized trial comparing n = 10 in the intervention group (foam rolling) to n = 10 controls. The study examined 13 outcome variables at 3 time points, but did not designate a primary outcome or timepoint and made no corrections for multiple testing. Using data from their Tables 1 and 2 [30], we have re-analyzed and re-interpreted the data for two variables: vertical jump height and quadriceps passive range of motion. Column 5 shows the p-values for the null hypothesis of no increase (H_0_: true effect≤0.2 SD), which corresponds to a one-sided minimal effects test. Using α = .05, we would fail to reject this null hypothesis for any outcome. Column 6 shows the p-values for the null hypothesis of decrease (H_0_: true effect≤-0.2 SD), which corresponds to a noninferiority test. Using α = .05, we would reject the null hypothesis for 4 of 6 outcomes. Though the paper concluded that foam rolling improved vertical jump height and passive range of motion, this re-analysis suggests that these conclusions were overly optimistic. At best the study could conclude that foam rolling was not detrimental to jump height or passive range of motion for some time points. Note that this re-analysis fails to account for the multiplicity of tests (39 total tests were run; only 6 are shown here).

^b^The p-values for the null hypothesis of no increase are obtained by subtracting the MBI benefit/positive probabilities from 1. For example, 1-.74 = .26.

^c^Effects were only deemed “clear” if the one-sided p-value for the null hypothesis of decrease was significant at p < .05 (the study used non-clinical MBI with *η*_1*b*_ = *η*_1*h*_ = 5%).

^d^This paper used a minimal evidence cutoff of “likely” for declaring substantial effects, specifying: “Results with a >75% likelihood were considered to be substantial.” [30]

^e^MBI probabilities were not given for “unclear” results, but we were able to back-calculate these p-values from the effect size estimate and 95% confidence intervals available in the paper.

In cases where papers do not report the exact MBI harm and benefit probabilities, it is possible to back-calculate the relevant p-values from confidence intervals. We include SAS and R code for doing this, specifically for between-group comparisons, in S2 Appendix. For ease of use, we have also created an online results converter (https://rehabinformatics.shinyapps.io/MBI_Converter/).

As a case-study, MBI has larger lessons for the statistical community. Some academics have suggested that moving researchers away from p-values and/or significance testing will improve applied statistical practice. MBI provides a natural experiment for testing this hypothesis. We found that MBI papers were no less prone to dichotomization or over-interpretation of findings; in fact, use of MBI generally led to greater exaggeration of results than standard significance testing. Other empirical investigations have drawn similar conclusions: A review of 31 papers published in *Basic and Applied Social Psychology* after the journal banned p-values found that many authors over-interpreted their results far beyond what the data supported [71]. Misuse of frequentist tools therefore appears to be a symptom of a deeper problem rather than the cause of bad statistics. To improve statistical practice in applied disciplines, we will have to address more fundamental problems such as poor statistical knowledge among applied researchers; an unsophisticated view of statistics as a black box set of recipes that provides simple answers; and endogenous and exogenous incentives to report “positive” findings.

## 5. Conclusions

We have conducted a systematic review of the MBI literature and undertaken mathematical analyses of MBI methods. We found that MBI has done direct harm to the sports science and medicine literature by causing authors to draw overly optimistic conclusions from their data. MBI has also promoted small studies, promulgated a simplistic “black box” approach to statistics, and reinforced the harmful view that the purpose of research is to get publishable results for the researcher. Sports scientists should stop using MBI. Past empirical studies that have used MBI may well include useful descriptive statistics, but their conclusions should be treated with skepticism. As a case-study, MBI has larger lessons for the statistical community: Our findings suggest that moving researchers away from significance testing and p-values does not improve applied statistical practice.

## Supporting information

S1 Appendix Details of the systematic review (including the assessment of bias and quality assurance checks), and details of the simulations and mathematics for estimating Type 1 error rates. (PDF)

S2 Appendix SAS and R code for implementing the simulations, and for converting MBI summary statistics into p-values for minimal effects testing. (PDF)

S3 Appendix Magnitude based inference spreadsheet for comparing the means of two groups. Implemented in Microsoft Excel. Obtained from www.sportsci.org in August 2019.
(XLS)

S1 File PRISMA 2009 checklist. (DOC)

S2 File (CSV)
