# Supplementary material for: Systematic review of the use of “magnitude-based inference” in sports science and medicine
Source: PLoS One. 2020 Jun 26;15(6):e0235318. doi: 10.1371/journal.pone.0235318 (PMC7319293; doi:10.1371/journal.pone.0235318)
Supplement: S1 Appendix — (PDF) [file pone.0235318.s001.pdf]

Supplemental Appendix I:
Systematic Review of the use of "Magnitude-Based Inference" in Sports Science and Medicine Papers

Keith R. Lohse, PhD[1,2], Kristin L. Sainani[3], J. Andrew Taylor[4], Michael L. Butson[5], Emma J. Knight[6], & Andrew J. Vickers[7*]

Systematic Review Methods: Data Extraction

A re-creation of the data extraction tool is shown in Supplemental Figure 1. Percentage agreement was moderate to high across items, median = 77%, IQR = [65%, 88%]. A third reviewer (KRL) then conducted consensus coding for all entries on all items in which there was disagreement. Remaining points of disagreement or areas of ambiguity where discussed with a fourth reviewer (KLS). Both the third and fourth reviewer were blind to the identities of the initial raters. For numeric variables in which there was a clear objective answer (e.g., total sample size, number of groups), the third and fourth reviewer discussed disagreement through to a single agreed upon value. For numeric variables in which there was not a clear objective answer (e.g., number of dependent variables, number of statistical tests run), disagreement was resolved by averaging the values extracted by the initial reviewers. We conducted a number of cross-tabulations to facilitate spot-checking and quality assurance of the data. For example, as shown in Supplementary Table 1, the primary statistical effect tested in each study aligned with the study design.

Supplemental Figure 1. Re-creation of the questions included in the data extraction tool. Each paper was reviewed by two randomly assigned authors and data were stored in a REDCap database.

MBI Systematic Review – Data Extraction Sheet V4

Indexing

**1. File Name from Box:**	
**2. Reviewer Initials:**	
**3. Journal Title:**	

Design information

**4. Total Number of Participants used in Analysis?**<br>**(Total N after exclusions.)**	
**5. Number of Groups:**<br>**(For between-subject variables only; otherwise leave blank.)**	
**6. Does the paper include a power analysis of any sort?**	
**7. Does the paper specify one dependent variable as "primary"?**	
**8. Which best describes the design of the study?**	
(Choose: RCT, cross-over study, observational, other)	
**9. Is the effect of primary interest between-subjects, within-subjects, or an interaction?**	
**11. If the effect of primary interest is an interaction, was it properly tested? (E.g., not a "here not there" comparison of t-tests)**	
**12. Estimated number of dependent variables:**	
**13. Was there any participant attrition and/or data exclusion?**	
**14. Drop-out refers to participants who left the study (for any reason) and therefore did not have data available.**<br><br>**How many drop-outs were there?**	
**15. Exclusions refer to participants whose data were available, but were excluded by the authors (for any reason).**<br><br>**How many people were excluded?**<br><br>**(In total, across all groups and exclusion criteria.)**	

Statistical Analysis

**16. Is the approached described as Bayesian in the manuscript?**	
**17. Is there an NHST for the primary hypothesis (along-side the MBI test)?**	
**18. If Yes, what was the alpha value for this test?**	
Details of the MBI analysis:	
**19. In the MBI details, what is the minimum threshold for HARM? (Enter the signed value.)**<br>(A common limit for the trivial threshold is -0.2.)	
**20. In the MBI details, what is the minimum threshold for BENEFIT? (Enter the signed value.)**<br>(A common limit for the trivial threshold is 0.2.)	
**21. In the MBI details, what criterion was used for the maximum risk of harm?**<br>(Common values are 0.5% or 5.0%, put NA if missing. We can often back-calculate this value if missing.)	
**22. In the MBI details, what criterion was used for the minimum chance of benefit?**<br>(Common values are 25% or 75%, put NA if missing.)	
**23. Did the authors explicitly state using clinical MBI or non-clinical MBI?**	

Conclusion and Interpretation

**24. In your view, is the conclusion in the Abstract written in a way that supports the hypotheses of the study?**<br>(Note this question is getting at "hypothesizing after results are known". We want to see if studies are unusually good at finding support for their stated hypotheses.)<br><br>(Yes, No, or there was no clear directional hypothesis)	
**24. How are effects interpreted in the Conclusion of the Abstract?**<br>These effects are usually a descriptor of certainty plus a magnitude (E.g., MBI recommends certain descriptors, such as "likely harm/benefit", "very likely trivial", or "certain harm/benefit".)<br><br>Authors may also describe their interventions as "substantial" or "implementable". Please insert all relevant descriptors separated by a semi-colon.)	

Risk of Bias Assessment

### Selection Bias	
**25. Selection Bias: Was there random assignment to groups/orders?**<br>This question only applies to interventions. For observational studies, put "not applicable."	
**26. Selection Bias: Is the method of randomization specified?**<br>This question only applies to interventions. For observational studies, put "not applicable."	
### Performance Bias	
**27. Performance Bias: Was there blinding to the intervention procedures?**<br>This question only applies to interventions. For observational studies, put "not applicable."	
**28. Performance Bias: Blinding in sport science studies is not always feasible, is there a statement that participants were blind to the hypotheses?**<br>This question applies to interventions and observational studies. For secondary analyses of collected data, put "Not Applicable."	
### Detection Bias	
**29. Detection Bias: Was there blinding of the outcome assessment?**<br>This question applies to all study designs.	
**30. Detection Bias: Is the outcome assessment sufficiently objective that blinding is unlikely to make a difference?**<br>This question applies to all study designs.	

Additional Comments

**31. Additional Comments:**<br>Please take note of any other oddities you found in the studies. For instance, some studies seem to confuse a clear observed difference in the sample with the inference to the population (i.e., "the treatment group might have performed better than control").	

Supplemental Table 1. Example cross-tabulations from the final dataset.

	Study Design			
**Hypothesized Effect**	**Cross-Over**	**Observational**	**RCT**	**Other**
Between-Subjects Main-Effect (#)	0	32	19	5
Within-Subjects Main-Effect (#)	48	35	0	6
Interaction (#)	10	14	34	8
Other (#)	0	14	0	7

MBI parameters (i.e., the trivial thresholds, maximum risk of harm/η_{1h}, and the minimum evidence threshold) were critical to the simulations undertaken in the main paper, so we also conducted a thorough review of those values. There was good agreement for the minimum threshold for harm (-δ) with the median value being -0.2 SD and the minimum threshold for benefit (+δ) with the median value being +0.2 SD. Similarly, there was moderate initial agreement between reviewers for the maximum risk of harm/η_{1h}, with the most common thresholds being 5% or 0.5% as shown in Supplemental Figure 2.

Coding for the minimum evidence threshold had generally lower agreement, because this was only explicitly stated in 23% of papers. An example of an explicit statement is found in Cruz et al. 2018[1]: "A likely difference (>75%) was considered as the minimum threshold to detect meaningful differences." Where this was not explicitly stated, we used the objective criterion of what results the authors presented as substantive in their abstract, conclusion section, and/or first paragraph of their discussion section. For example, Lahart et al. 2018[2] write in their abstract: "Magnitude-based inference analyses revealed **likely** at least small beneficial effects (effect sizes≥.20) on absolute and relative VO_2max (d=.44 and .40, respectively), and total and moderate PA (d= .73 and .59, respectively) in the intervention compared to the usual care group. We found no **likely** beneficial improvements in any other outcome" (emphasis added). They then recommend the intervention's use: "This intervention has the potential for widespread implementation and adoption, which could considerably impact on post-treatment recovery in this population." One can infer from their description and ensuing recommendation that they used

“likely” as their minimum evidence threshold, though this was never explicitly stated. As another example, Weston et al. (2015)[3] write in their abstract that, “Compared to the control group, the core training intervention group had a **possibly** large beneficial effect on 50-m swim time” and then say, “This is the first study to demonstrate a **clear beneficial effect** of isolated core training on 50-m front crawl swim performance” (emphasis added). The authors implicitly used “possible” as their minimum evidence threshold, because they draw definitive conclusions from a “possible” effect. To ensure consistency in the coding of this parameter, reviewers 3 or 4 reviewed all 232 papers to confirm the reported values.

As shown in Supplemental Figure 2, the median threshold for evidence was 50%, but this was not the most common value, with most studies using a threshold of 75% (n=100) or 25% (n=88). In a few cases, this choice was ambiguous and was coded as unclear (n = 25).

Supplemental Figure 2. The ‘Maximum Risk of Harm’ and the ‘Threshold for Evidence’ across studies.

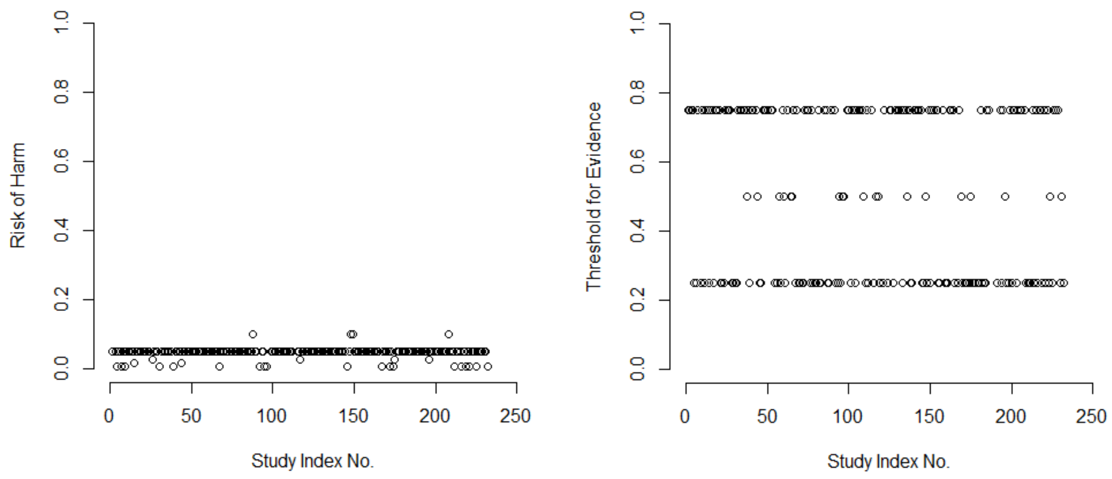

Systematic Review Methods: Risk of Bias Assessment

To assess the potential risk of bias in our data, we had two questions related specifically to Selection Bias, Performance Bias, and Detection Bias in our data extraction tool. Other questions about

attrition, data exclusions, and the reporting of outcomes tap into potential Attrition Bias and Reporting Bias (Higgins & Green, 2011)[4]. Descriptive statistics related to each type of bias are presented below, but our primary focus was on the risk of Selection, Performance, and Detection Bias in these studies.

Detection Bias. Detection bias was assessed by two questions that asked about (1) random assignment to groups/conditions and (2) if the method of randomization was explicit. As shown in Supplemental Table 2, most controlled trials and cross-over designs had random assignment to groups/conditions but very few studies were explicit about the method of randomization.

Supplemental Table 2. Summary of questions relating to detection bias as a function of study design.

	**Study Design**			
**Random Assignment?**	**Cross-Over**	**Observational**	**Other**	**RCT**
-- No	7	3	6	4
-- Not Applicable	0	89	14	0
-- Yes	51	3	6	49
**Explicit Method?**				
-- No	46	5	10	35
-- Not Applicable	4	91	15	0
-- Yes	8	0	1	18

Performance Bias. Performance bias was assessed by two questions that asked if (1) participants were blinded to intervention procedures/conditions and (2) because blinding is not always feasible, if there was an explicit statement that participants were naïve to the hypotheses the experiment. As shown in Supplemental Table 3, blinding of participants was not common except in cross-over designs. Furthermore, it was not common for authors to explicitly state that participants were naïve to the hypotheses of the experiment.

Supplemental Table 3. Summary of questions relating to performance bias as a function of study design.

	**Study Design**			
**Blinding of Participants?**	**Cross-Over**	**Observational**	**Other**	**RCT**
-- No	31	9	13	40

-- Not Applicable	0	86	9	0
-- Yes	27	0	4	13
**Naïve to Hypotheses?**				
-- No	51	33	18	52
-- Not Applicable	0	61	6	0
-- Yes	7	1	2	1

Detection Bias. Detection bias was assessed by two questions that asked if (1) assessors were blinded to intervention procedures/conditions and (2) were the outcomes measures sufficiently objective that assessor blinding was unlikely to make a difference (in the judgment of the raters). As shown in Supplemental Table 4, blinding of assessors was rarely stated across study types. This concern is somewhat ameliorated, however, by the frequency of objective measures of performance.

Supplemental Table 4. Summary of questions relating to detection bias as a function of study design.

	**Study Design**			
**Blinding of Assessors?**	**Cross-Over**	**Observational**	**Other**	**RCT**
-- No	40	63	18	40
-- Not Applicable	0	32	5	0
-- Yes	18	0	3	13
**Sufficiently Objective?**				
-- Mixed	18	18	3	14
-- No	18	14	7	14
-- Not Applicable	0	32	5	0
-- Yes	22	31	11	25

Note that we added a category of "mixed" for the objectivity of measures because a subset of the dependent variables was judged to be sufficiently objective whereas others were not.

Attrition Bias. Of the 232 studies included in the review, 55 (24%) reported some degree of attrition or exclusion of data. Among those studies that reported dropout, the median number of participants dropping out of the study was 5, IQR[2, 9]. Among those studies that reported exclusion of existing data the median number of participants excluded was 4, IQR[1, 10]. Given the sample sizes in these studies, this amount of attrition is worrisome (the median total sample size for studies reporting

attrition was 21, IQR[16, 30]). It is worth noting that these exclusions were only reported in 24% of all studies, but among those studies that reported attrition, it was common for up to 25% of the data to be censored or excluded.

Reporting Bias. Reporting bias is difficult to discern, but in our data extraction tool reviewers were asked to rate if the abstract was, "written in a way that supported the hypotheses" of a given study. For 104 cases (45%), this was rated as not clear because no specific directional hypothesis was given. In cases where hypothesis support was clear, the abstract supported the hypotheses in 72 cases (31%), clearly did not support the hypotheses in 8 cases (3%), and presented mixed results in 37 cases (16%). An additional 11 cases (5%) were rated at "Other". The lack of clear directional hypotheses in most cases suggests that a substantial number of these studies were exploratory in nature. Assuming that authors are reporting all measures they collected, however, we do not think that the risk of reporting bias is higher in these studies than in other studies in the field.

Estimation of the Type I Error Rates for Magnitude-Based Inference

Simulations

We simulated the Type I error rates for MBI for both a between-group comparison and a within-group comparison assuming a range of sample sizes. For the between-group comparison, we generated 200,000 simulated trials with n per group from two normally distributed populations with the same variance and zero or a trivial difference between the groups. For the within-group comparison, we generated 200,000 simulated trials with a sample size of n from a normally distributed population with a true effect size of 0 or a trivial effect size. Type I error rates were then calculated as the percentage of studies in which MBI returned a positive or negative inference that met a given minimum evidence threshold (e.g., "likely") or the percentage of studies where $p<.05$ (for standard hypothesis testing). For Figures 2C and 2D, we simulated 5000 between-group trials and identified MBI inferences as:

- "Likely" or higher effect: (LCL90 ≥ -δ and LCL50 ≥ δ) or (UCL90 ≤ δ and UCL50 ≤ -δ)
- "Possible" effect: (LCL90 ≥ -δ and UCL50 ≥ δ and LCL50 < δ) or (UCL90 ≤ δ and LCL50 ≤ -δ and UCL50 ≥ -δ)
- "Trivial" effect: (LCL90 ≥ -δ and UCL50 ≤ δ) or (UCL90 ≤ δ and LCL50 ≥ -δ)
- "Unclear" effect: LCL90 ≤ -δ and UCL90 ≥ δ

Simulations were conducted in SAS 9.4. See Supplemental Appendix II for the simulation code; we have also provided code for the same simulations in R (see Supplemental Appendix II).

Mathematical Calculations

We also calculated the Type I error rates mathematically. Mathematical symbols used:

n=sample size in each group

η_1 = significance level for deciding "unclear"

η_2 = significance level for the minimum evidence threshold of interest

δ_h = threshold for harm

δ_b = threshold for benefit

$T_1 = T_{(1-\eta_1),2n-2}$

$T_2 = T_{\eta_2,2n-2}$

ES= true effect size

Z represents the standard normal distribution

χ_{2n-2} is the chi-square distribution with 2n-2 degrees of freedom

Sainani[5] previously showed that to achieve a minimum evidence threshold for benefit in clinical MBI, one must meet two constraints: (1) p<η_{1h} for H_0: true effect ≤ -δ_h; otherwise, the inference would be deemed "unclear" and (2) p<η_2 for H_0: true effect ≤ δ_b, where η_2 is the significance threshold that determines the exact inference achieved, whether "possibly", "likely", etc. η_2 =.05 corresponds to "very likely"; η_2 =.25 corresponds to "likely"; and η_2 =.75 corresponds to "possibly." For example, to achieve a clear beneficial inference of at least "likely" when η_{1h} =5%, one must meet:

1. p<. 05 for H_0: true effect ≤ -δ_h (constraint on harm)

2. p<.25 for H_0: true effect ≤ δ_b (constraint on benefit)

Sainani[5] previously derived equations for these two constraints, for the problem of comparing two means. For simplicity, we use a pooled sample variance, s^2, and equal sample sizes for the groups. The two constraints are:

$T_1 = T_{(1-\eta_1),2n-2}$

$T_2 = T_{\eta_2,2n-2}$

1. observed value $\geq (-\delta_h + T_1 \times \sqrt{\frac{2s^2}{n}})$
2. observed value $\geq (\delta_b - T_2 \times \sqrt{\frac{2s^2}{n}})$

Thus, to meet a given minimum evidence threshold for benefit, the following must be true:

$$\text{observed value} \geq \max((-\delta_h + T_1 \times \sqrt{\frac{2s^2}{n}}), (\delta_b - T_2 \times \sqrt{\frac{2s^2}{n}}))$$

We have extended this to accommodate both directions, i.e., non-clinical MBI. For non-clinical MBI, a minimum evidence threshold for harm or benefit is met when:

$$\text{observed value} > 0 \text{ and observed value} \geq \max((-\delta_h + T_1 \times \sqrt{\frac{2s^2}{n}}), (\delta_b - T_2 \times \sqrt{\frac{2s^2}{n}}))$$

or

$$\text{observed value} < 0 \text{ and observed value} \leq \min((-\delta_h + T_2 \times \sqrt{\frac{2s^2}{n}}), (\delta_b - T_1 \times \sqrt{\frac{2s^2}{n}}))$$

A Type I error occurs when the above conditions are met but the true effect is 0 or trivial. (Note that, for the sake of comparison between MBI and traditional methods, we count a statistically significant result as a Type I error if the true effect size is trivial but non-zero.) Sainani[5] previously derived an approximate equation for the Type I error rate of clinical MBI. We have adapted this equation to accommodate non-clinical MBI and to provide an exact solution by integrating over the sample variance:

Type I error probability (where -δ_h≤ES≤ δ_b)=

$$\int_0^\infty \mathrm{P}(Z > \max((\frac{-\delta_h+T_1\times\sqrt{\frac{2(x\sigma^2/(2n-2))}{n}}-ES}{\sqrt{\frac{2\sigma^2}{n}}}),(\frac{\delta_b-T_2\times\sqrt{\frac{2(x\sigma^2/(2n-2))}{n}}-ES}{\sqrt{\frac{2\sigma^2}{n}}}))\, p(\chi_{2n-2}=x)dx +$$

$$\int_0^\infty \mathrm{P}(Z < \min((\frac{-\delta_h+T_2\times\sqrt{\frac{2(x\sigma^2/(2n-2))}{n}}-ES}{\sqrt{\frac{2\sigma^2}{n}}}),(\frac{\delta_b-T_1\times\sqrt{\frac{2(x\sigma^2/(2n-2))}{n}}-ES}{\sqrt{\frac{2\sigma^2}{n}}}))\, p(\chi_{2n-2}=x)dx$$

Math-predicted and simulated results matched perfectly, as shown in Supplemental Figure 3. See Supplemental Appendix II for SAS and R code that implements the math equations. Note that this equation gives the probability of achieving a given inference. This probability is a Type I error rate when the true effect size is 0 or trivial, but is a Type II error rate when the true effect is non-trivial.

Supplemental Figure 3. Math-predicted (blue lines) versus simulated (red lines) Type I error rates. Lines exactly overlap. Simulations used n=200,000 trials. Settings: trivial range of -0.2 to 0.2; η_1= 5%; non-clinical MBI; true effect=0; variance of 0.364 for pre-post parallel trial; and 1.0 for cross-sectional comparison.

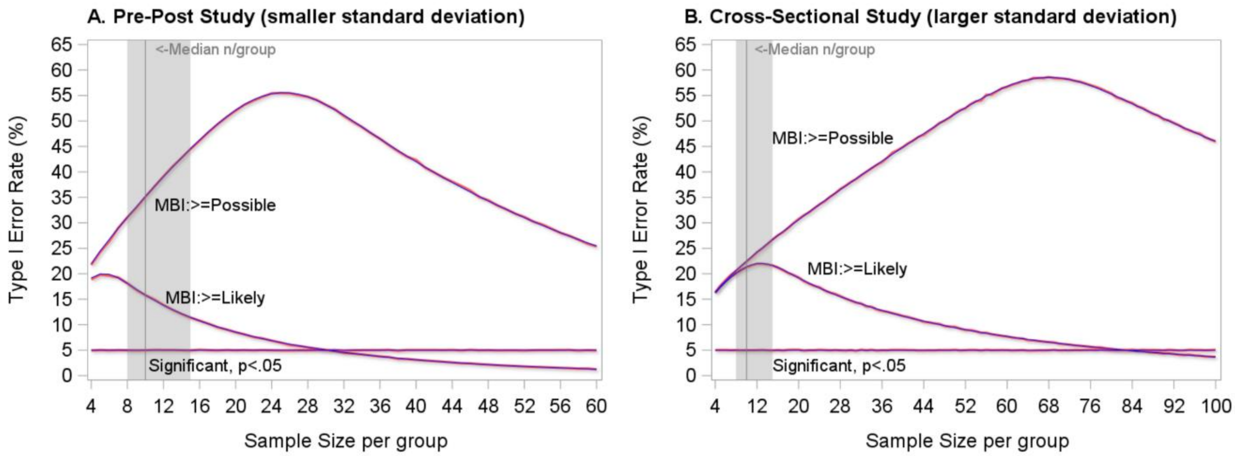

Results from the between-group comparison are shown visually within the main paper. Results for a within-person study design are shown in Supplemental Figure 4.

Supplemental Figure 4. Type I error rates (mathematically calculated) for a within-person study for MBI's "possible" (purple) and "likely" (red) thresholds. True effect size = 0. Standard hypothesis testing with a significance level of 0.05 is shown in blue. The shaded area is the interquartile range of total sample sizes among within-person studies. Typical MBI settings were used (thresholds for harm/benefit

of -0.2/0.2; η_1= 5%; and equivalent treatment of positive and negative directions). Assumes within-person variances of 0.364 (A,B) and 0.80 (C) and trivial range of 0.2 (A,C) and 0.1 (B) between-person standard deviations. In papers we reviewed, within-person studies sometimes used smaller trivial ranges (B) and typically had large within-person variance (C).

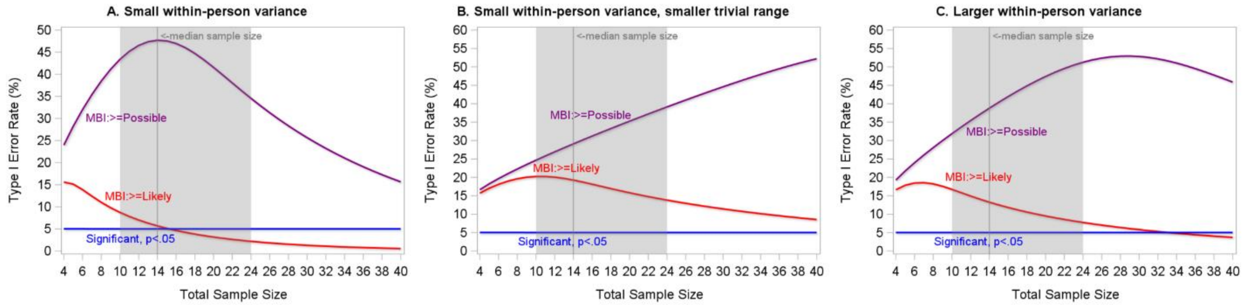

We calculated Type I error rates for a range of scenarios (Table 3 of the main paper). All reported values were confirmed by both simulation and math. For the base-case, we assumed a η_1= 5%; and thresholds of harm/benefit of 0.2 standard deviations; these were varied in some simulations. Effect sizes were expressed in standard deviation units; the true effect was set at 0 or a trivial amount (e.g., 0.1). For studies that assumed a cross-sectional comparison, we assumed that the variance was 1.0. For studies that assumed a pre-post parallel trial design, we allowed the within-person variance to differ from the between-person variance; in the base case, we set this at 0.364 (assumes pre-post correlation is r=.818).

References

1. Lahart IM, Carmichael AR, Nevill AM, Kitas GD, Metsios GS. The effects of a home-based physical activity intervention on cardiorespiratory fitness in breast cancer survivors; a randomised controlled trial. *J Sports Sci.* 2018*;*36:1077-1086.

2. de Freitas Cruz, I., Pereira, L. A., Kobal, R., Kitamura, K., Cedra, C., Loturco, I., & Abad, C. C. C. (2018). Perceived training load and jumping responses following nine weeks of a competitive period in young female basketball players. *PeerJ*, 6, e5225.

3. Weston M, Hibbs AE, Thompson KG, Spears IR. Isolated core training improves sprint performance in national-level junior swimmers. *International Journal of Sports Physiology and Performance*. 2015; 10: 204-210.

4. Higgins JPT, Green, S. *Cochrane handbook for systematic reviews of interventions*. 2011. The Cochrane Collaboration.

5. Sainani KL. The Problem with" Magnitude-based Inference". *Med Sci Sports Exerc*. 2018;50:2166-2176.
