# Supplementary material for: Systematic review of the use of “magnitude-based inference” in sports science and medicine
Source: PLoS One. 2020 Jun 26;15(6):e0235318. doi: 10.1371/journal.pone.0235318 (PMC7319293; doi:10.1371/journal.pone.0235318)
Supplement: S2 Appendix — (PDF) [file pone.0235318.s002.pdf]

Supplemental Appendix II: SAS and R code

Keith R. Lohse, PhD, Kristin L. Sainani, J. Andrew Taylor, Michael L. Butson, Emma J. Knight, & Andrew J. Vickers

Sections:

1. SAS code for simulating Type I error rates
2. SAS code for mathematically calculating Type I error rates
3. R code for simulating Type I error rates
4. R code for mathematically calculating Type I error rates
5. SAS code for converting MBI results into minimum effects tests
6. R code for converting MBI results into minimum effects tests

1. SAS code for simulating Type I error rates (Figure 2, Figure 3, Supplemental Figure 3)

```
/*Analyst: Kristin Sainani
/*August 2019*/
/*This code estimates Type I error rates for MBI using simulation*/
/*This code is a simplified version of code provided in Sainani KL.
"The Problem with Magnitude-Based Inference" MSSE 2018.
/*1. Estimates Type I error rates as a function of sample size for a
two-group comparison (two-sample ttest)
/*2. Estimates Type I error rates as a function of sample size for a
within-group comparison (paired ttest)
/*Results match those generated by the more complicated simulations as
well as the mathematically predicted results*/

/*1. Simulation based on two sample t-test, between-group comparison--
for pre-post study, variance may be smaller than 1*/
/*Simulations are meant to generate values for graphing Type I error
against sample size*/
%LET NoOfTrials=200000; *number of repeats;
%LET Var=1; *variance, use 1 for cross-sectional study. May be less
than 1 for pre-post study;
%LET StdzedMagniThresh=.2; *threshold for harm and benefit;
%LET ES=0; *True effect size, must be in the trivial range for Type I
error;

/*STEP 1: Generate the simulated data*/
data data;
do n=6 to 60 by 1;
do Trial=1 to &NoOfTrials;
do athlete=1 to n;
  Group="A";
  DeltaY=0+sqrt(&Var)*rannor(0); *change score for pre-post study or
abs value for cross-sectional study;
```

```
  output;
  end;
do athlete=1 to n;
  Group="B";
  DeltaY=&ES+sqrt(&Var)*rannor(0);
  output;
  end;
end;
end;
run;
/*STEP 2: Generate relevant statistics from each simulated trial*/
proc means data=data mean std noprint; *output appropriate summary
statistics for each trial;
var deltaY;
output out=out mean=estimate std=std n=n;
by n trial group;
run;
data dat2;
set out;
estimateA=estimate;
stdA=std;
where group="A";
keep n trial stdA estimateA;
run;
data dat3;
set out;
estimateB=estimate;
stdB=std;
where group="B";
keep n trial stdB estimateB;
run;
data dat4;
merge dat2 dat3;
by n trial;
run;
data dat4;
set dat4;
estimate=estimateB-estimateA;
std=sqrt((stdA**2+stdB**2)/2); *pooled variance for equal sample
sizes;
run;
data estimates; *generate confidence limits needed to call inferences;
set dat4;
StdzedMagniThresh=&StdzedMagniThresh;
df=2*n-2;
ES=&ES;
err=sqrt(2*std**2/n);
tvalue=abs(estimate/err);
probt=2*(probt(-tvalue,df));
t50=Tinv(1-.25,df);
t99=Tinv(1-.005,df);
t90=Tinv(1-.05,df);
```

```
ucl50=estimate+t50*err;
ucl90=estimate+t90*err;
ucl99=estimate+t99*err;
lcl50=estimate-t50*err;
lcl90=estimate-t90*err;
lcl99=estimate-t99*err;
run;
/*STEP 3: Call the inferences*/
data errors; *calculates the probability of finding a likely,
possible, or significant positive effect and a likely, possible, or
significant negative effect.
Clinical MBI "implementable" is also included;
set estimates;
psigpMBI1=0; *Probability of concluding "likely" positive effect;
psignMBI1=0; *probability of concluding "likely" negative effect;
psigpMBI2=0; *probability of concluding "possible" positive effect;
psignMBI2=0; *probability of concluding "possible" negative effect;
psigpMBI3=0; *probability of concluding "implementable" positive
effect;
psignMBI3=0; *probability of conclusing "implementable" negative
effect--doesn't exist in clinical MBI;
psigpHT=0; *probability of concluding significant positive result;
psignHT=0; *probability of concluding significant negative result;
  if estimate>0 and (LCL90>-StdzedMagniThresh and
LCL50>StdzedMagniThresh) then psigpMBI1=100;
  if estimate>0 and (UCL50>StdzedMagniThresh and LCL90>-
StdzedMagniThresh) then psigpMBI2=100;
  if estimate>0 and (UCL50>StdzedMagniThresh and LCL99>-
StdzedMagniThresh) then psigpMBI3=100;
  if estimate>0 and probt<.05 then psigpHT=100;
  if estimate<0 and (UCL50<-StdzedMagniThresh and
UCL90<StdzedMagniThresh) then psignMBI1=100;
  if estimate<0 and (LCL50<-StdzedMagniThresh and
UCL90<StdzedMagniThresh) then psignMBI2=100;
  if estimate<0 and (LCL50<-StdzedMagniThresh and
UCL99>StdzedMagniThresh) then psignMBI3=100;
  if estimate<0 and probt<.05 then psignHT=100;
run;

/*STEP 4: Calculate the probabilities of different inferences for each
ES/sample size combination*/
proc sort data=errors; by n ; run;
proc means data=errors mean  noprint ; *Calculate the probabilities of
finding likely, possible, significant results by effect size and
sample size;
var psigpMBI1 psignMBI1 psigpMBI2 psignMBI2 psigpMBI3 psignMBI3
psigpHT psignHT ;
output out=Probabilities mean=psigpMBI1 psignMBI1 psigpMBI2 psignMBI2
psigpMBI3 psignMBI3 psigpHT psignHT;
by n ;
run;
data errors;
```

```
set probabilities;
typeIm1=psigpMBI1+psignMBI1;
typeIm2=psigpMBI2+psignMBI2;
typeImbi3=psigpMBI3+psignMBI3;
typeIht=psigpht+psignht;
keep typeIm1 typeIm2 typeImbi3 typeIht n;
run;

proc print data=errors;
var n typeIm1 typeIm2 typeIht;
run;



/*2. Simulation based on paired t-test, within-group comparison*/
/*Simulations are meant to generate graphs of Type I error against
sample size*/
%LET NoOfTrials=25000; *number of repeates;
%LET Var=0.8; *within-person variance;
%LET StdzedMagniThresh=.2; *thresholds for harm/benefit;
%LET ES=0; *True effect size, must be in trivial range for Type I
error;

/*STEP 1: Generate the simulated data*/
data dat;
do n=4 to 60 by 1;
do Trial=1 to &NoOfTrials;
Group="A";
do athlete=1 to n;
  DeltaY=&ES+sqrt(&Var)*rannor(0);
  output;
  end;
end;
end;
run;
/*STEP 2: Generate relevant statistics from each simulated trial*/
proc means data=dat mean std noprint; *output appropriate summary
statistics for each trial;
var deltaY;
output out=out mean=estimate std=std n=n;
by n trial;
run;

data estimates; *generate values needed;
set out;
StdzedMagniThresh=&StdzedMagniThresh;
df=n-1;
ES=&ES;
err=std/sqrt(n);
tvalue=abs(estimate/err);
probt=2*(probt(-tvalue,df));
t50=Tinv(1-.25,df);
```

```
t99=Tinv(1-.005,df);
t90=Tinv(1-.05,df);
ucl50=estimate+t50*err;
ucl90=estimate+t90*err;
ucl99=estimate+t99*err;
lcl50=estimate-t50*err;
lcl90=estimate-t90*err;
lcl99=estimate-t99*err;
run;
/*STEP 3: Call the inferences*/
data errors; *calculates the probability of finding a likely,
possible, or significant positive effect and a likely, possible, or
significant negative effect
(also includes clinical MBI "implementable");
set estimates;
psigpMBI1=0; *Probability of concluding "likely" positive effect;
psignMBI1=0; *probability of concluding "likely" negative effect;
psigpMBI2=0; *probability of concluding "possible" positive effect;
psignMBI2=0; *probability of concluding "possible" negative effect;
psigpMBI3=0; *probability of concluding "implementable" positive
effect;
psignMBI3=0; *probability of conclusing "implementable" negative
effect--doesn't exist in clinical MBI;
psigpHT=0; *probability of concluding significant positive result;
psignHT=0; *probability of concluding significant negative result;
  if estimate>0 and (LCL90>-StdzedMagniThresh and
LCL50>StdzedMagniThresh) then psigpMBI1=100;
  if estimate>0 and (UCL50>StdzedMagniThresh and LCL90>-
StdzedMagniThresh) then psigpMBI2=100;
  if estimate>0 and (UCL50>StdzedMagniThresh and LCL99>-
StdzedMagniThresh) then psigpMBI3=100;
  if estimate>0 and probt<.05 then psigpHT=100;
  if estimate<0 and (UCL50<-StdzedMagniThresh and
UCL90<StdzedMagniThresh) then psignMBI1=100;
  if estimate<0 and (LCL50<-StdzedMagniThresh and
UCL90<StdzedMagniThresh) then psignMBI2=100;
  if estimate<0 and (LCL50<-StdzedMagniThresh and
UCL99>StdzedMagniThresh) then psignMBI3=100;
  if estimate<0 and probt<.05 then psignHT=100;
run;

/*STEP 4: Calculate the probabilities of different inferences for each
ES/sample size combination*/
proc sort data=errors; by n ES ; run;
proc means data=errors mean  noprint ; *Calculate the probabilities of
finding likely, possible, significant results by effect size and
sample size;
var psigpMBI1 psignMBI1 psigpMBI2 psignMBI2 psigpMBI3 psignMBI3
psigpHT psignHT ;
output out=Probabilities mean=psigpMBI1 psignMBI1 psigpMBI2 psignMBI2
psigpMBI3 psignMBI3 psigpHT psignHT;
by n ES ;
```

```
run;
data prob2;
set probabilities;
typeImbi1=psigpMBI1+psignMBI1;
typeImbi2=psigpMBI2+psignMBI2;
typeImbi3=psigpMBI3+psignMBI3;
typeIht=psigpht+psignht;
typeIht2=psigpht2+psignht2;
typeIbayes=psigpbayes+psignbayes;
keep n typeImbi1 typeImbi2 typeImbi3 typeIht;
run;
proc print data=prob2;
run;
```

2. SAS code for mathematically calculating Type I error rates (Figure 2, Supplemental Figures 3 and 4)

```
/**Type I error calculator for non-clinical MBI and NHST**/
/*Analyst: Kristin Sainani*/
/*August 2019*/
/*Reference: Formulas and code for the TypeI and TypeII error rates of
clinical MBI come from: Sainani KL. "The Problem with Magnitude Based
Inference." MSSE 2018.*/
/*I have made two changes from the original code:
/*1. Adaptation to Non-Clinical MBI: Published equations were for
Clinical MBI, which is a one-sided test.  Non-clinical MBI is the same
except
it implements two one-sided test. Thus, I adapted the formulas to
incorporate two one-sided tests.*/
/*2. I have added an integration routine over the sample variance to
allow an exact solution for Type I error.

/*The code below generates the Type I error rates as a function of:
1. Sample size
2. Effect size (must be in the trivial range for Type I error)
3. Variance (choosing SD=1 for cross-sectional study and SD=0.364 to
1.0 for pre-post study)
4. MBI parameters
-thresholds for harm and benefit (typically -0.2/0.2)
-maximum risk of harm (typically 5%)
-minimum chance of benefit: =25% for the "possible" evidence threshold
and 75% for the "likely" evidence threshold
/*For NHST, I used the fact that NHST=MBI when threshold for
harm/benefit~0 to calculate the rates*/
/*I confirmed the mathematical results through simulation (see code
document 1).*/

/*Calculator 1: Type I error rates for two-group parallel trial (pre-
post or cross-sectional), based on a two-sample ttest*/
```

```
%LET var=0.364; *the specific value of 0.364 is from a pre-post design
with r=.818. Std dev at baseline=1.0.
Variance in change scores=1+1-2*.818=.364. For cross-sectional study,
set the variance to 1.0;
%LET deltab=0.2; *threshold for benefit;
%LET deltah=0.2; *threshold for harm;
%LET maxrisk=.05; *maximum risk of harm;
%LET ES=0; *effect size. Must be in the trivial range for Type I
error;
%LET thresh1=.75; *MBI evidentiary threshold of interest, eg
"likely"=.75;
%LET thresh2=.25; *second MB evidentiary threshold of interest;

data errors;
do n=5 to 60 by 1; *range of sample sizes desired;
df=2*n-2; *degrees of freedom;
err=sqrt(2)*sqrt(&var)/sqrt(n); *true standard error;
th=Tinv((1-&maxrisk),df); *T for harm, e.g. 1.64 for 90% CI;
tb1=Tinv(1-&thresh1,df); *T for benefit, assuming eg 75% minimum
chance benefit (MBI, likely threshold);
tb2=Tinv(1-&thresh2,df); *T for benefit, assuming eg 25% minimum
chance benefit (MBI, possible threshold);
thht=Tinv((1-.025),df); *T for harm, standard hypothesis testing,
using the fact that MBI collapses to HT when threshold ~0 to calculate
rates for HT, .05 significance level;
TypeIm1=0; *Stores the Type I error for MBI threshold one;
TypeIm2=0; *Stores the Type I error for MBI threshold two;
TypeIht=0; *Stores the Type I error standard HT;
do estvar=.001 to 3*&var by .001; *integrate by parts over the range
of possible sample variances, incrementing every .001;
esterr=sqrt(estvar*2/n); *sample standard error;
hterm=-&deltah+th*esterr; *constraint on harm;
bterm1=&deltab-tb1*esterr; *constraint on benefit, threshold 1;
bterm2=&deltab-tb2*esterr; *constraint on benefit, threshold 2;
htermht=-.0001+thht*esterr; *constraint on harm for standard
hypothesis testing. Harm term dominates in all cases for standard HT;
pVar=probchi((df*(estvar+.001)/&var),df)-probchi((df*estvar/&var),df);
*probability of each sample variance in .001 increments;
pPositivep1=1-probnorm(((max(hterm,bterm1)-&ES)/err)); *the
probability of reaching
the given MBI threshold (eg "clear, >=75% beneficial"). The observed
value must be greater than the maximum
of the harm and benefit constraints;
pPositiven1=1-Probnorm((max(hterm,bterm1)+&ES)/err); *the probability
of reaching
the given MBI threshold on the negative side;
pPositivep2=1-Probnorm((max(hterm,bterm2)-&ES)/err); *repeat for
second MBI threshold;
pPositiven2=1-Probnorm((max(hterm,bterm2)+&ES)/err);
pPositivehtp=1-Probnorm((htermht-&ES)/err); *repeat for standard HT
(constraint term always bigger);
```

```
pPositivehtn=1-Probnorm((htermht+&ES)/err);
TypeIm1=(pPositiven1+pPositivep1)*pVar*100+TypeIm1;*integrate over all
possible sample variances to get the probability
of incurring the specific MBI inference (e.g., ">=75% beneficial" for
each sample size;
TypeIm2=(pPositiven2+pPositivep2)*pVar*100+TypeIm2;
TypeIht=(pPositivehtn+pPositivehtp)*pVar*100+TypeIht;
end;
output; *output the Type I error for each sample size;
end;
run;

proc print data=errors;
var n TypeIm1 TypeIm2 TypeIht;
run;

/*Calculator 2: Type I error rates for a within-group comparison,
based on paired t-test*/

%LET var=.364; *true variance;
%LET deltab=0.2; *threshold for benefit;
%LET deltah=0.2; *threshold for harm;
%LET maxrisk=.05; *maximum risk of harm;
%LET ES=0; *effect size. Must be in the trivial range for Type I
error;
%LET thresh1=.75; *MBI evidentiary threshold of interest, eg
"likely"=.75;
%LET thresh2=.25; *second MB evidentiary threshold of interest;

data errors;
do n=5 to 60 by 1; *range of sample sizes desired;
df=n-1; *degrees of freedom;
err=sqrt(&var)/sqrt(n); *true standard error for paired ttest;
th=Tinv((1-&maxrisk),df); *T for harm, e.g. 1.64 for 90% CI;
tb1=Tinv(1-&thresh1,df); *T for benefit, assuming eg 75% minimum
chance benefit (MBI, likely threshold);
tb2=Tinv(1-&thresh2,df); *T for benefit, assuming eg 25% minimum
chance benefit (MBI, possible threshold);
thht=Tinv((1-.025),df); *T for harm, standard hypothesis testing,
using the fact that MBI collapses to HT when threshold ~0 to calculate
rates for HT, .05 significance level;
TypeIm1=0; *Stores the Type I error for MBI threshold one;
TypeIm2=0; *Stores the Type I error for MBI threshold two;
TypeIht=0; *Stores the Type I error standard HT;
do estvar=.001 to 3*&var by .001; *integrate by parts over the range
of possible sample variances, incrementing every .001;
esterr=sqrt(estvar)/sqrt(n); *sample standard error;
hterm=-&deltah+th*esterr; *constraint on harm;
bterm1=&deltab-tb1*esterr; *constraint on benefit, threshold 1;
bterm2=&deltab-tb2*esterr; *constraint on benefit, threshold 2;
htermht=-.0001+thht*esterr; *constraint on harm for standard
hypothesis testing. Harm term dominates in all cases for standard HT;
```

```
pVar=probchi((df*(estvar+.001)/&var),df)-probchi((df*estvar/&var),df);
*probability of each sample variance in .001 increments;
pPositivep1=1-probnorm(((max(hterm,bterm1)-&ES)/err)); *the
probability of reaching
the given MBI threshold (eg "clear, >=75% beneficial"). The observed
value must be greater than the maximum
of the harm and benefit constraints;
pPositiven1=1-Probnorm((max(hterm,bterm1)+&ES)/err); *the probability
of reaching
the given MBI threshold on the negative side;
pPositivep2=1-Probnorm((max(hterm,bterm2)-&ES)/err); *repeat for
second MBI threshold;
pPositiven2=1-Probnorm((max(hterm,bterm2)+&ES)/err);
pPositivehtp=1-Probnorm((htermht-&ES)/err); *repeat for standard HT
(constraint term always bigger);
pPositivehtn=1-Probnorm((htermht+&ES)/err);
TypeIm1=(pPositiven1+pPositivep1)*pVar*100+TypeIm1;*integrate over all
possible sample variances to get the probability
of incurring the specific MBI inference (e.g., ">=75% beneficial" for
each sample size;
TypeIm2=(pPositiven2+pPositivep2)*pVar*100+TypeIm2;
TypeIht=(pPositivehtn+pPositivehtp)*pVar*100+TypeIht;
end;
output; *output the Type I error for each sample size;
end;
run;

proc print data=errors;
var n TypeIm1 TypeIm2 TypeIht;
run;
```

3. R code for simulating Type I error rates

```
# Keith Lohse
# 2019-08-29
# Adapted from Kristin Sainani (2019)
library("ggplot2"); library("car"); library("dplyr")

## Simulation for Independent Samples T-Test ----
## Simulating a Data Set ----
set.seed(17)

NoOfTrials<-2000
Var<-0.36
StdzedMagniThresh<-.2
ES<-0

# Generating a simulated data frame
n<-c(seq(from=5, to=60, by=1))
trial<-c(seq(from=1, to=NoOfTrials, by=1))

index<-c(seq(from =1, to=length(n)*NoOfTrials, by=1))
```

```
sim_data<-data.frame(index)

head(sim_data)

for (j in 1:length(n)) {
  group_n <- n[j]
  print(j)

  for (i in 1:length(trial)){
    SampleA<-rnorm(n=group_n, mean=0, sd=sqrt(Var))
    SampleB<-rnorm(n=group_n, mean=ES, sd=sqrt(Var))

    sim_data$ES[NoOfTrials*(j-1)+i] <- ES
    sim_data$n_per_group[NoOfTrials*(j-1)+i] <- group_n
    sim_data$groupA_mean[NoOfTrials*(j-1)+i] <- mean(SampleA)
    sim_data$groupB_mean[NoOfTrials*(j-1)+i] <- mean(SampleB)
    sim_data$groupA_sd[NoOfTrials*(j-1)+i] <- sd(SampleA)
    sim_data$groupB_sd[NoOfTrials*(j-1)+i] <- sd(SampleB)
  }
}

summary(as.factor(sim_data$n_per_group))


## Summary Statistics ----
sim_data$mean_diff <- sim_data$groupB_mean-sim_data$groupA_mean
boxplot(sim_data$mean_diff~sim_data$n_per_group)

# Pooled variance for equal sample sizes
sim_data$s_pooled <- sqrt(((sim_data$groupA_sd^2)+(sim_data$groupB_sd^2))/2)

sim_data$df <- (2*sim_data$n_per_group)-2
sim_data$SE <- sqrt(2*((sim_data$s_pooled^2)/sim_data$n_per_group))
sim_data$t_obs <- sim_data$mean_diff/sim_data$SE
boxplot(sim_data$t_obs~sim_data$n_per_group)

sim_data$p_null <- 2*pt(abs(sim_data$t_obs), df=sim_data$df,
lower.tail=FALSE)
boxplot(sim_data$p_null~sim_data$n_per_group)
sim_data$sig_null <- as.factor(sim_data$p_null<0.05)
sim_data$sig_null<-recode(sim_data$sig_null,
                          "FALSE"="p>0.05",
                          "TRUE" ="p<0.05")

# Note that the Type 1 Error Rate is consistent across effect sizes.

sim_data$ucl50=sim_data$mean_diff+sim_data$SE*qt(1-.25, df=sim_data$df)
sim_data$ucl90=sim_data$mean_diff+sim_data$SE*qt(1-.05, df=sim_data$df)
sim_data$ucl99=sim_data$mean_diff+sim_data$SE*qt(1-.005, df=sim_data$df)

sim_data$lcl50=sim_data$mean_diff-sim_data$SE*qt(1-.25, df=sim_data$df)
sim_data$lcl90=sim_data$mean_diff-sim_data$SE*qt(1-.05, df=sim_data$df)
sim_data$lcl99=sim_data$mean_diff-sim_data$SE*qt(1-.005, df=sim_data$df)

## MBI Inferences
# Clear versus Unclear
sim_data$clear<- as.factor(ifelse((sim_data$ucl90 > StdzedMagniThresh &
```

```
                            sim_data$lcl90 < (-1*StdzedMagniThresh)),
                         "unclear", "clear"))

summary(sim_data$clear)
xtabs(~sig_null+clear, data=sim_data)

# Likelihoods
sim_data$vlb<-as.numeric(sim_data$lcl90 > StdzedMagniThresh &
                           sim_data$clear == "clear")
summary(as.factor(sim_data$vlb))

sim_data$lb<-as.numeric(sim_data$lcl50 > StdzedMagniThresh &
                          sim_data$clear == "clear")
summary(as.factor(sim_data$lb))

sim_data$pb<-as.numeric(sim_data$ucl50 > StdzedMagniThresh &
                          sim_data$clear == "clear")
summary(as.factor(sim_data$pb))

sim_data$pt<-as.numeric(sim_data$ucl50 < StdzedMagniThresh &
                          sim_data$lcl50 > (-1*StdzedMagniThresh) &
                          sim_data$clear == "clear")
summary(as.factor(sim_data$pt))

sim_data$vlt<-as.numeric(sim_data$ucl90 < StdzedMagniThresh &
                           sim_data$lcl90 > (-1*StdzedMagniThresh) &
                          sim_data$clear == "clear")
summary(as.factor(sim_data$vlt))

sim_data$ph<-as.numeric(sim_data$lcl50 < (-1*StdzedMagniThresh) &
                          sim_data$clear == "clear")
summary(as.factor(sim_data$ph))

sim_data$lh<-as.numeric(sim_data$ucl50 < (-1*StdzedMagniThresh) &
                          sim_data$clear == "clear")
summary(as.factor(sim_data$lh))

sim_data$vlh<-as.numeric(sim_data$ucl90 < (-1*StdzedMagniThresh) &
                          sim_data$clear == "clear")
summary(as.factor(sim_data$vlh))


head(sim_data, 10)
sim_data$mbi_inference<-as.factor(paste(sim_data$clear, sim_data$vlb,
                              sim_data$lb, sim_data$pb,
                              sim_data$pt, sim_data$vlt,
                              sim_data$ph, sim_data$lh, sim_data$vlh,
                              sep=""))
summary(sim_data$mbi_inference)

sim_data$mbi_inference<-recode(sim_data$mbi_inference,
                               unclear00000000="Unclear",
                               clear00000111="Very Likely Harmful",
                               clear00000110="Likely Harmful",
                               clear00000100="Possibly Harmful",
                               clear00011000="Very Likely Trivial",
                               clear00010000="Possibly Trivial",
```

```
                                          clear00100000="Possibly Beneficial",
                                          clear01100000="Likely Beneficial",
                                          clear01100000="Likely Beneficial",
                                          clear11100000="Very Likely Beneficial")

summary(sim_data$mbi_inference)

head(sim_data)
xtabs(~n_per_group+mbi_inference, data=sim_data)

write.csv(sim_data, "./sim_data_between_var036.csv")

## Plotting MBI Inferential Results ----
# Ridges Graph
library(ggridges)

n_of_10<-subset(sim_data, n_per_group==10)
n_of_20<-subset(sim_data, n_per_group==20)
n_of_60<-subset(sim_data, n_per_group==60)

g1<- ggplot(n_of_10, aes(x = mean_diff, y = mbi_inference, fill =
mbi_inference)) +
    geom_density_ridges(scale=1.5, alpha=0.7, rel_min_height = 0.01)

g2<-g1+scale_x_continuous(name = "Mean Difference", limits=c(-2,2)) +
  scale_y_discrete(name = "MBI Inference") +
  ggtitle("n/group = 10")

g3 <- g2 + theme(axis.text=element_text(size=14, colour="black"),
        axis.title=element_text(size=14,face="bold")) +
  theme(strip.text.x = element_text(size = 14))+
  theme(legend.position="none")

plot(g3)

# Scatter
g1<- ggplot(n_of_10, aes(x = mean_diff, y = index,
                         fill = mbi_inference)) +
  geom_point(pch=21, alpha=0.5, size=2)

g2<-g1+scale_x_continuous(name = "Mean Difference", limits=c(-2,2)) +
  scale_y_discrete(name = "Simulation Number") +
  ggtitle("n/group = 10")

g3 <- g2 + theme(axis.text=element_text(size=14, colour="black"),
                 axis.title=element_text(size=14,face="bold")) +
  theme(strip.text.x = element_text(size = 14))
  #theme(legend.position="none")

plot(g3)



## Simulation for Dependent Samples T-Test ----
## Simulating a Data Set ----
set.seed(17)
```

```
NoOfTrials<-2000
Var_within<-0.36 # Within-Person Variance of the difference
# The Within-Person variance can also be calculated from the between-person
# variance if the correlation between repeated measures is known:
# Var_within = var_pooled*(1-r)

StdzedMagniThresh<-.2
ES<-0

# Generating a simulated data frame
n<-c(seq(from=6, to=60, by=1))
trial<-c(seq(from=1, to=NoOfTrials, by=1))

index<-c(seq(from =1, to=length(n)*NoOfTrials, by=1))
sim_data<-data.frame(index)

head(sim_data)

for (j in 1:length(n)) {
  group_n <- n[j]
  print(j)

  for (i in 1:length(trial)){
    print(paste(j, i))

    SampleA<-rnorm(n=group_n, mean=0, sd=sqrt(Var_within))

    sim_data$ES[NoOfTrials*(j-1)+i] <- ES
    sim_data$n_per_group[NoOfTrials*(j-1)+i] <- group_n
    sim_data$mean_diff[NoOfTrials*(j-1)+i] <- mean(SampleA)
    sim_data$sd_diff[NoOfTrials*(j-1)+i] <- sd(SampleA)
    }
}

summary(as.factor(sim_data$n_per_group))



## Summary Statistics ----
summary(sim_data$mean_diff)
boxplot(sim_data$mean_diff~sim_data$n_per_group)

# SE of the difference
sim_data$df <- sim_data$n_per_group-1
sim_data$SE_diff <- sim_data$sd_diff/sqrt(sim_data$n_per_group)
sim_data$t_obs_within <- sim_data$mean_diff/sim_data$SE_diff
boxplot(sim_data$t_obs_within~sim_data$n_per_group)

sim_data$p_null <- 2*pt(abs(sim_data$t_obs_within),
                        df=sim_data$df, lower.tail=FALSE)

boxplot(sim_data$p_null~sim_data$n_per_group)
sim_data$sig_null <- as.factor(sim_data$p_null<0.05)
sim_data$sig_null<-recode(sim_data$sig_null,
                          "FALSE"="p>0.05",
                          "TRUE" ="p<0.05")
```

```
# Note that the Type 1 Error Rate is consistent across effect sizes.

sim_data$ucl50=sim_data$mean_diff+sim_data$SE_diff*qt(1-.25, df=sim_data$df)
sim_data$ucl90=sim_data$mean_diff+sim_data$SE_diff*qt(1-.05, df=sim_data$df)
sim_data$ucl99=sim_data$mean_diff+sim_data$SE_diff*qt(1-.005, df=sim_data$df)

sim_data$lcl50=sim_data$mean_diff-sim_data$SE_diff*qt(1-.25, df=sim_data$df)
sim_data$lcl90=sim_data$mean_diff-sim_data$SE_diff*qt(1-.05, df=sim_data$df)
sim_data$lcl99=sim_data$mean_diff-sim_data$SE_diff*qt(1-.005, df=sim_data$df)

## MBI Inferences
# Clear versus Unclear
sim_data$clear<- as.factor(ifelse((sim_data$ucl90 > StdzedMagniThresh &
                                    sim_data$lcl90 < (-
1*StdzedMagniThresh)),
                                 "unclear", "clear"))

summary(sim_data$clear)
xtabs(~sig_null+clear, data=sim_data)

# Likelihoods
sim_data$vlb<-as.numeric(sim_data$lcl90 > StdzedMagniThresh &
                           sim_data$clear == "clear")
summary(as.factor(sim_data$vlb))

sim_data$lb<-as.numeric(sim_data$lcl50 > StdzedMagniThresh &
                          sim_data$clear == "clear")
summary(as.factor(sim_data$lb))

sim_data$pb<-as.numeric(sim_data$ucl50 > StdzedMagniThresh &
                          sim_data$clear == "clear")
summary(as.factor(sim_data$pb))

sim_data$pt<-as.numeric(sim_data$ucl50 < StdzedMagniThresh &
                          sim_data$lcl50 > (-1*StdzedMagniThresh) &
                          sim_data$clear == "clear")
summary(as.factor(sim_data$pt))

sim_data$vlt<-as.numeric(sim_data$ucl90 < StdzedMagniThresh &
                           sim_data$lcl90 > (-1*StdzedMagniThresh) &
                           sim_data$clear == "clear")
summary(as.factor(sim_data$vlt))

sim_data$ph<-as.numeric(sim_data$lcl50 < (-1*StdzedMagniThresh) &
                          sim_data$clear == "clear")
summary(as.factor(sim_data$ph))

sim_data$lh<-as.numeric(sim_data$ucl50 < (-1*StdzedMagniThresh) &
                          sim_data$clear == "clear")
summary(as.factor(sim_data$lh))

sim_data$vlh<-as.numeric(sim_data$ucl90 < (-1*StdzedMagniThresh) &
                           sim_data$clear == "clear")
summary(as.factor(sim_data$vlh))


head(sim_data, 10)
```

```
sim_data$mbi_inference<-as.factor(paste(sim_data$clear, sim_data$vlb,
                                        sim_data$lb, sim_data$pb,
                                        sim_data$pt, sim_data$vlt,
                                        sim_data$ph, sim_data$lh,
sim_data$vlh,
                                        sep=""))
summary(sim_data$mbi_inference)

sim_data$mbi_inference<-recode(sim_data$mbi_inference,
                               unclear00000000="Unclear",
                               clear00000111="Very Likely Harmful",
                               clear00000110="Likely Harmful",
                               clear00000100="Possibly Harmful",
                               clear00011000="Very Likely Trivial",
                               clear00010000="Possibly Trivial",
                               clear00100000="Possibly Beneficial",
                               clear01100000="Likely Beneficial",
                               clear01100000="Likely Beneficial",
                               clear11100000="Very Likely Beneficial")

summary(sim_data$mbi_inference)

xtabs(~mbi_inference+sig_null, data=sim_data)

write.csv(sim_data, "./sim_data_within_v036_marg01.csv")



## Plotting MBI Inferential Results ----
# Ridges Graph
library(ggridges)

n_of_10<-subset(sim_data, n_per_group==10)
n_of_20<-subset(sim_data, n_per_group==20)
n_of_60<-subset(sim_data, n_per_group==60)

g1<- ggplot(n_of_10, aes(x = mean_diff, y = mbi_inference, fill =
mbi_inference)) +
  geom_density_ridges(scale=1.5, alpha=0.7, rel_min_height = 0.01)

g2<-g1+scale_x_continuous(name = "Mean Difference", limits=c(-1,1)) +
  scale_y_discrete(name = "MBI Inference") +
  ggtitle("n = 10 (Within Subject)")

g3 <- g2 + theme(axis.text=element_text(size=14, colour="black"),
                 axis.title=element_text(size=14,face="bold")) +
  theme(strip.text.x = element_text(size = 14))+
  theme(legend.position="none")

plot(g3)

# Scatter
g1<- ggplot(n_of_10, aes(x = mean_diff, y = index,
                         fill = mbi_inference)) +
  geom_point(pch=21, alpha=0.5, size=2)

g2<-g1+scale_x_continuous(name = "Mean Difference", limits=c(-2,2)) +
```

```
  scale_y_discrete(name = "Simulation Number") +
  ggtitle("n = 10 (Within Subject)")

g3 <- g2 + theme(axis.text=element_text(size=14, colour="black"),
                 axis.title=element_text(size=14,face="bold")) +
  theme(strip.text.x = element_text(size = 14))
#theme(legend.position="none")

plot(g3)
```

4. R code for mathematically calculating Type I error rates

```
# Keith Lohse
# 2019-08-29
# Adapted from Kristin Sainani (2019)
library("ggplot2"); library("car"); library("dplyr")

## Calculator #1: Simulation for Independent Samples T-Test ----
## Simulating a Data Set ----
set.seed(17)

var <- 0.364 # Between-person (within group) variance for an independent
samples design
# The Within-Person variance (i.e., of the difference) can also be calculated
# from the between-person variance if the correlation between repeated
measures
# is known.
# Var_within = var_pooled*(1-r)

deltab <- 0.2 # threshold for benefit
deltah <- 0.2 # threshold for harm
maxrisk <- 0.05 # maximum risk of harm
ES <- 0.0 # effect size, must be in the trivial range for a Type 1 error.

thresh1<-0.75 # MBI evidentiary threshold of interest, eg "likely"=.75
thresh2<-0.25 # second MB evidentiary threshold of interest, eg "possible" =
0.25

# Generating a simulated data frame
n<-c(seq(from=5, to=60, by=1))


estvar <- seq(from=.001, to = (3*var), by = .001)
# integrate by parts over the range of possible sample variances,
# incrementing every .001


index<-c(seq(from =1, to=length(n), by=1))
sim_errors<-data.frame(index)

head(sim_errors)
c <- 0

for (j in 1:length(n)) {
```

```
  c <- c + 1
  print(c)
  sim_errors$group_n[j]<-n[j]
  df <- 2*n[j]-2 # degrees of freedom for independent samples t-test
  err <- sqrt(2)*sqrt(var)/sqrt(n[j]) # standard error for independent
samples t-test

  th=qt((1-maxrisk),df) # T for harm, e.g. 1.64 for 90% CI
  tb1=qt(1-thresh1,df) # T for benefit, assuming eg 75% minimum chance
benefit (MBI, likely threshold)
  tb2=qt(1-thresh2,df) # T for benefit, assuming eg 25% minimum chance
benefit (MBI, possible threshold);
  thht=qt((1-.025),df) # T for harm, standard hypothesis testing,
  # using the fact that MBI collapses to HT when threshold ~0 to calculate
  # rates for HT, .05 significance level

  TypeIm1=0 # Stores the Type I error for MBI threshold one;
  TypeIm2=0 # Stores the Type I error for MBI threshold two;
  TypeIht=0 # Stores the Type I error standard HT;

  for (i in 1:length(estvar)){
    esterr=sqrt(estvar[i]*2/n[j]) # sample standard error;

    hterm=(-deltah)+th*esterr # constraint on harm
    bterm1=deltab-tb1*esterr # constraint on benefit, threshold 1;
    bterm2=deltab-tb2*esterr # constraint on benefit, threshold 2;
    htermht=(-.0001)+thht*esterr # constraint on harm for standard hypothesis
testing.
    # Harm term dominates in all cases for standard HT

    pVar=pchisq((df*(estvar[i]+.001)/var),df)-pchisq((df*estvar[i]/var),df)
    # probability of each sample variance in .001 increments;
    pPositivep1=1-pnorm(((max(hterm,bterm1)-ES)/err));
    # the probability of reaching the given MBI threshold
    #(eg "clear, >=75% beneficial"). The observed value must be greater than
the
    # maximum of the harm and benefit constraints
    pPositiven1=1-pnorm((max(hterm,bterm1)+ES)/err) # the probability of
reaching
    # the given MBI threshold on the negative side
    pPositivep2=1-pnorm((max(hterm,bterm2)-ES)/err)
    # repeat for second MBI threshold;
    pPositiven2=1-pnorm((max(hterm,bterm2)+ES)/err)
    pPositivehtp=1-pnorm((htermht-ES)/err)
    # repeat for standard HT (constraint term always bigger)
    pPositivehtn=1-pnorm((htermht+ES)/err)

    TypeIm1=(pPositiven1+pPositivep1)*pVar*100+TypeIm1
    # integrate over all possible sample variances to get the probability
    # of incurring the specific MBI inference (e.g., ">=75% beneficial" for
each sample size;
    TypeIm2=(pPositiven2+pPositivep2)*pVar*100+TypeIm2;
    TypeIht=(pPositivehtn+pPositivehtp)*pVar*100+TypeIht;
  }
  sim_errors$TypeIm1[c]= TypeIm1
  sim_errors$TypeIm2[c]= TypeIm2
  sim_errors$TypeIht[c]= TypeIht
```

```
}

head(sim_errors, 20)

plot(TypeIm1~group_n, pch=21, bg="purple", data=sim_errors, ylim=c(0,100),
     main=paste("Between-Subjects, var = ", var))
points(TypeIm2~group_n, pch=21, bg="firebrick", data=sim_errors)
points(TypeIht~group_n, pch=21, bg="dodgerblue", data=sim_errors)

var(sim_errors$TypeIm1)
var(sim_errors$TypeIm2)
var(sim_errors$TypeIht)








## Calculator #2: Simulation for Dependent Samples T-Test ----
## Simulating a Data Set ----
set.seed(17)

var <- 0.364 # Variation of the difference.
# The Within-Person variance (i.e., of the difference) can also be calculated
# from the between-person variance if the correlation between repeated
measures
# is known.
# Var_within = var_pooled*(1-r)

deltab <- 0.2 # threshold for benefit
deltah <- 0.2 # threshold for harm
maxrisk <- .05 # maximum risk of harm
ES <- 0 # effect size, must be in the trivial range for a Type 1 error.

thresh1<-0.75 # MBI evidentiary threshold of interest, eg "likely"=.75
thresh2<-0.25 # second MB evidentiary threshold of interest, eg "possible" =
0.25

# Generating a simulated data frame
n<-c(seq(from=5, to=60, by=1))


estvar <- seq(from=.001, to = (3*var), by = .001)
# integrate by parts over the range of possible sample variances,
# incrementing every .001

index<-c(seq(from =1, to=length(n), by=1))
sim_errors<-data.frame(index)

head(sim_errors)
c <- 0

for (j in 1:length(n)) {
  c <- c + 1
  print(c)
  sim_errors$group_n[j]<-n[j]
```

```
  df <- n[j]-1 # degrees of freedom for independent samples t-test
  err <- sqrt(var)/sqrt(n[j]) # standard error for independent samples t-test

  th=qt((1-maxrisk),df) # T for harm, e.g. 1.64 for 90% CI
  tb1=qt(1-thresh1,df) # T for benefit, assuming eg 75% minimum chance
benefit (MBI, likely threshold)
  tb2=qt(1-thresh2,df) # T for benefit, assuming eg 25% minimum chance
benefit (MBI, possible threshold);
  thht=qt((1-.025),df) # T for harm, standard hypothesis testing,
  # using the fact that MBI collapses to HT when threshold ~0 to calculate
  # rates for HT, .05 significance level

  TypeIm1=0 # Stores the Type I error for MBI threshold one;
  TypeIm2=0 # Stores the Type I error for MBI threshold two;
  TypeIht=0 # Stores the Type I error standard HT;

  for (i in 1:length(estvar)){
    esterr=sqrt(estvar[i])/sqrt(n[j]) # sample standard error;

    hterm=(-deltah)+th*esterr # constraint on harm
    bterm1=deltab-tb1*esterr # constraint on benefit, threshold 1;
    bterm2=deltab-tb2*esterr # constraint on benefit, threshold 2;
    htermht=(-.0001)+thht*esterr # constraint on harm for standard hypothesis
testing.
    # Harm term dominates in all cases for standard HT

    pVar=pchisq((df*(estvar[i]+.001)/var),df)-pchisq((df*estvar[i]/var),df)
    # probability of each sample variance in .001 increments;
    pPositivep1=1-pnorm(((max(hterm,bterm1)-ES)/err));
    # the probability of reaching the given MBI threshold
    #(eg "clear, >=75% beneficial"). The observed value must be greater than
the
    # maximum of the harm and benefit constraints
    pPositiven1=1-pnorm((max(hterm,bterm1)+ES)/err) # the probability of
reaching
    # the given MBI threshold on the negative side
    pPositivep2=1-pnorm((max(hterm,bterm2)-ES)/err)
    # repeat for second MBI threshold;
    pPositiven2=1-pnorm((max(hterm,bterm2)+ES)/err)
    pPositivehtp=1-pnorm((htermht-ES)/err)
    # repeat for standard HT (constraint term always bigger)
    pPositivehtn=1-pnorm((htermht+ES)/err)

    TypeIm1=(pPositiven1+pPositivep1)*pVar*100+TypeIm1
    # integrate over all possible sample variances to get the probability
    # of incurring the specific MBI inference (e.g., ">=75% beneficial" for
each sample size;
    TypeIm2=(pPositiven2+pPositivep2)*pVar*100+TypeIm2;
    TypeIht=(pPositivehtn+pPositivehtp)*pVar*100+TypeIht;
  }
  sim_errors$TypeIm1[c]= TypeIm1
  sim_errors$TypeIm2[c]= TypeIm2
  sim_errors$TypeIht[c]= TypeIht
}

head(sim_errors, 20)
```

```
plot(TypeIm1~group_n, pch=21, bg="purple", data=sim_errors, ylim=c(0,100),
     main=paste("Within-Subject, var = ", var))
points(TypeIm2~group_n, pch=21, bg="firebrick", data=sim_errors)
points(TypeIht~group_n, pch=21, bg="dodgerblue", data=sim_errors)


var(sim_errors$TypeIm1)
var(sim_errors$TypeIm2)
var(sim_errors$TypeIht)
```

5. SAS code for converting MBI results into minimum effects tests.

```
/*Analyst: Kristin Sainani*/
/*Date: April 2020*/
/*MACRO to extract standard errors and p-values from a confidence
interval for a mean difference*/
/*GOAL: When given the confidence interval for a difference in two
independent means, this MACRO returns standard errors, as well as p-
values for two specific hypothesis tests:
1. two-sided mull hypothesis significance testing (NHST), H0:0
2. one-sided minimal effects test against a positive or negative
smallest effect of interest (SESOI).
This MACRO can be used to reanalyze data from MBI studies. */
/*USER INPUTS:
1. n1: size of group 1
2. n2: size of group 2
3. conflevel: confidence level of the confidence interval, entered as
a whole number, e.g., 90
4. lowerCL: lower confidence limit
5. upperCL: upper confidence limit
6. SESOI: smallest effect size of interest, must be given in the same
units as the upper and lower confidence limits and entered as an
absolute value (note: if confidence interval is given in non-
standardized units, then the SESOI must be given in
non-standardized units).
Assumptions: Assumes that the confidence interval is for a comparison
of two independent means and was calculated assuming equal variances
Explanation: Confidence Interval = point estimate +/- T*standard error
Standard error = (half width of confidence interval)/T
Note: **Answers may be affected by rounding in the reported
estimates**/

%macro convert(n1=, n2=, conflevel=, lowerCL=, upperCL=, SESOI=);
data convert;
df=(&n1.+&n2.-2); *degrees of freedom assuming pooled variance was
used;
confleveld=&conflevel/100; *confidence level as a decimal;
Tvalue=-tinv(((1-confleveld)/2),df); *find the T-value used to
construct the CI;
```

```
halfwidth=(&upperCL.-&lowerCL.)/2; *find half the width of the
confidence interval;
meandiff=&lowerCL.+halfwidth; *find the point estimate;
stderr=halfwidth/Tvalue; *find the standard error;
label stderr="Standard Error for the mean difference";
label meandiff="Observed difference in means";
TNHST=abs(meandiff/stderr); *T-value for testing H0=0;
pNHST=2*probT(-TNHST, df); *p-value for two-sided NHST;
label pNHST="p-value for two-sided NHST";
TMETp=(meandiff-&SESOI.)/stderr; *T-value for testing Ho<=+SESOI;
pMETp=probT(-TMETp, df); *p-value for one-sided minimal effects test
against positive SESOI;
label pMETp="p-value for one-sided minimal effects test, positive
direction";
TMETn=(meandiff+&SESOI.)/stderr; *T-value for testing Ho>=-SESOI;
pMETn=probT(TMETn, df); *p-value for one-sided minimal effects test
against positive SESOI;
label pMETn="p-value for one-sided minimal effects test, negative
direction";
T95=-tinv((.05/2),df);
lower95=meandiff-T95*stderr;
label lower95="lower limit of the 95% confidence interval";
upper95=meandiff+T95*stderr;
label upper95="upper limit of the 95% confidence interval";
run;
proc print data=convert label noobs;
var stderr pNHST pMETp pMETn lower95 upper95;
run;
%mend;
/*Example implementation, uses post-24 vertical jump data from
MacDonald et al. Med Sci Sports Exerc. 2014;46: 131-142.
Standardized values are entered*/
%convert(n1=10, n2=10, conflevel=90, lowerCL=-0.4, upperCL=1.43,
SESOI=0.2);
```

6. R code for converting MBI results into minimum effects tests.

```
# Title: MBI to MET/TOST Converter
# Author: Keith Lohse, PhD, PStat
# Date: 2020-05-04

# Writing the conversion function:
convert<-function(n1, n2, conflevel, lowerCL, upperCL, SESOI) {
  df=(n1+n2-2) # degrees of freedom assuming pooled variance was used;
  confleveld=conflevel/100 # confidence level as a decimal;
  Tvalue=-qt(((1-confleveld)/2),df) # find the T-value used to construct the
CI;
  halfwidth=(upperCL-lowerCL)/2 # find half the width of the confidence
interval;
  meandiff=lowerCL+halfwidth # find the point estimate;
  stderr=halfwidth/Tvalue # find the standard error;
```

```
  TNHST=abs(meandiff/stderr) # T-value for testing H0=0;
  pNHST=2*pt(-TNHST, df) # p-value for two-sided NHST;

  TMETp=(meandiff-SESOI)/stderr # T-value for testing Ho<=+SESOI;
  pMETp=pt(-TMETp, df) # p-value for one-sided minimal effects test against
positive SESOI;

  TMETn=(meandiff+SESOI)/stderr  #T-value for testing Ho>=-SESOI;
  pMETn=pt(TMETn, df) # p-value for one-sided minimal effects test against
positive SESOI;

  T95=-qt((.05/2),df)
  lower95=meandiff-T95*stderr
  upper95=meandiff+T95*stderr

  labels = c("Standard Error for the mean difference",
             "Observed difference in means",
             "p-value for two-sided NHST",
             "p-value for one-sided minimal effects test, positive
direction",
             "p-value for one-sided minimal effects test, negative
direction",
             "lower limit of the 95% confidence interval",
             "upper limit of the 95% confidence interval")

  values = c(stderr, meandiff, pNHST, pMETp, pMETn, lower95, upper95)

  results<-data.frame(labels, values)
  print(results)
}


# Implementing the conversion function:
convert(n1=10, # Enter the sample sizes:
        n2=10,
        conflevel=90, # Enter your confidence level (as a percentage):
        lowerCL = -0.4, # Enter the confidence limits:
        upperCL = 1.43,
        SESOI = 0.2 # Smallest Effect Size of Interest
)
```
